# Photoresponsive Luminescent Silica Nanoparticles as Additive for 3D Printing and Electrospinning

**DOI:** 10.1002/asia.202401415

**Published:** 2024-12-12

**Authors:** Rick Y. Lorberg, Sidharth Thulaseedharan Nair Sailaja, Fabian Terlau, Maria Victoria Cappellari, Marco Schmiedtchen, Anzhela Galstyan, Cristian A. Strassert, Michael Giese, Jens Voskuhl

**Affiliations:** ^1^ Faculty of Chemistry (Organic Chemistry) Center of Medical Biotechnology (ZMB) Center for Nanointegration Duisburg-Essen (CENIDE) University of Duisburg-Essen Universitätsstraße 7 Essen 45117 Germany; ^2^ Faculty of Chemistry Center for Nanointegration Duisburg-Essen (CENIDE), Center of Medical Biotechnology (ZMB) and Center for Water and Environment Research (ZWU) University of Duisburg-Essen Universitätsstraße 5 Essen 45141 Germany; ^3^ Institute for Inorganic and Analytical Chemistry University of Münster Corrensstraße 28/30 Münster 48149 Germany; ^4^ CiMIC, SoN, CeNTech University of Münster Heisenbergstraße 11 Münster 48149 Germany; ^5^ GUIDE^Plus^ Co-Creation Lab Produktinnovationen (CCLP) Schützenbahn 70 Essen 45127 Germany

**Keywords:** Mesoporous silica nanoparticles, Luminophores, Photoreaction, Additive manufacturing, Electrospinning

## Abstract

In this study, we present the synthesis and a versatile way to incorporate photoresponsive organic luminophores into polymeric materials using mesoporous silica nanoparticles (MSNs). The encapsulated thioethers within the MSNs were employed in polyvinyl alcohol (PVA) films, resin‐based stereolithography, and electrospinning. Due to light‐induced cyclisation to dibenzothiophenes (DBTs), ***mm*** **OC_12_** loaded materials were used to inscribe images using UV light. The DBTs formed from ***mm*** **OC_12_** (***mm*** **DBT_A/B_**) exhibit a long phosphorescence afterglow, which was investigated by steady‐state and time‐resolved photoluminescence spectroscopy. In addition, scanning electron microscopy (SEM) imaging, including energy dispersive X‐ray spectroscopy (EDX), revealed the well‐dispersed and intact MSNs in the polymeric materials. This approach shows a general way to incorporate non‐polar organic luminophores into polymeric materials while retaining their unique emission properties.

## Graphical abstract

Luminescent nanoparticles were prepared by incorporating luminophores into silica nanoparticles in a Stöber‐like synthesis. These nanoparticles show good compatibility with additive manufacturing and electrospinning and can serve as a linkage to combine weakly soluble luminophores with aqueous media or polar polymers while maintaining the properties of the luminophore. Furthermore, ultralong lifetimes could be achieved in polymers and at 77 K.

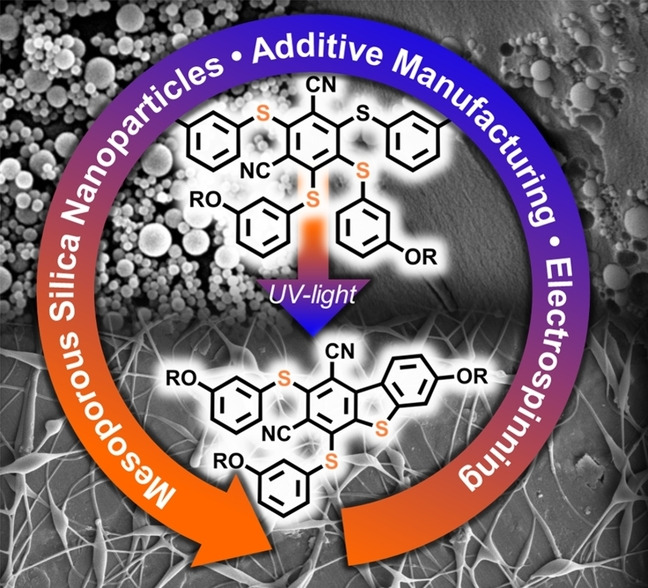

## Introduction

The unique functional properties and low weight of porous ceramics enable various technological applications, such as aerosol filters, biomaterials, catalyst supports, gas burner media, gas sensors, heat exchangers, porous electrodes, refractory materials, and thermal insulation.[^1^, ^2^, ^3^, ^4^] In general, the shaping of ceramic components involves powder mixtures and conventional methods such as moulding, sacrificial templates, and direct foaming.[^5^, ^6^, ^7^, ^8^, ^9^, ^10^] However, the brittleness and cost involved in manufacturing ceramics with complex forms were found to be challenging.^[11]^ To address these issues, an efficient alternative method called additive manufacturing (AM), also known as 3D printing, was developed. Through accurate layer‐by‐layer deposition, this fabrication technology makes it easier to manufacture highly complex components with customised geometries and multifunctional surface features.[^9^, ^10^] In addition, AM can translate digital models into complex 3D structures with high precision and accuracy. These approaches help to improve material efficiency by reducing waste generation and designing intricate geometries that are difficult to obtain through conventional manufacturing.^[12]^ Therefore, AM is a decisive technology that could transform manufacturing methodologies across various industries.

Recently, combining nanomaterials such as metallic nanoparticles,^[13]^ quantum dots,[^14^, ^15^, ^16^] graphene,^[17]^ fullerenes,^[18]^ carbon nanotubes,^[13]^ and nanofibers^[19]^ with AM to create novel composites and applications has gained significant popularity. Among them, mesoporous silica nanoparticles have emerged as a prominent alternative because of their large pore volumes, high surface areas, and versatile properties.[^20^, ^21^, ^22^]

Although luminescent materials have been widely applied in fields such as sensors, anti‐counterfeiting devices, and biomedicine, their use in 3D printing is stillscarce.[^23^, ^24^] This limitation in stereolithography (SLA) arises primarily from the poor miscibility of luminescent molecules with the resin matrix in the formulation and the quenching during the photopolymerisation, due to the competition between the light absorption of the added fluorophores and the photoinitiator, which interferes with the 3D printing procedures and also leads to unwanted side products.[^25^, ^26^] Furthermore, most of the reported studies have focused on commercially available fluorescent dyes, such as rhodamine derivatives, which are prone to photodegradation.[^24^, ^27^, ^28^] Therefore, a reliable methodology needs to be developed to incorporate versatile fluorophores with AM for 3D printing applications.

Moreover, AM electrospinning is a state‐of‐the‐art technology to create continuous, uniform nanofibers (NFs) and is known for its scalability and cost‐effectiveness.[^29^, ^30^] In the electrospinning process, a polymer solution is carefully fed through a fine metal syringe nozzle towards a conductive metal target.^[31]^ The resulting polymer fibers can vary in diameter, length, and alignment based on factors such as polymer solution properties, applied voltage, syringe tip size, and distance between the nozzle and the collector. Electrospinning has a history that stretches back almost a century.[^32^, ^33^] This process has evolved significantly over the decades, becoming a crucial method in various research areas and industrial applications due to the easy fabrication of nanosized materials.^[34]^ The resulting materials have been used in various fields such as biomedical engineering,^[35]^ drug delivery,^[36]^ textiles,^[37]^ filtration,^[38]^ and nanotechnology.^[39]^ Electrospun nanofibers containing fluorescent compounds have recently attracted attention and offer promising potential for a variety of applications in different fields, including sensors, photonics, forensics and medicine.[^40^, ^41^]

In this contribution, the very recently described photocyclisation of thioethers to DBTs^[42]^ was combined with MSN encapsulation and the fabrication of different polymeric materials using thin film formation, stereolithographic 3D printing (SLA) and electro‐spinning.

Two luminophores with dodecyloxy‐moieties (***mm*** **OC_12_** and ***pp*** **OC_12_**) were synthesised to ensure entrapment in the MSN via the well‐known *Stöber* process.[^43^, ^44^] The obtained MSNs with two distinct photo‐responsive luminophores were incorporated into materials and investigated in depth regarding their dispersibility, photophysics as well as application as photoresponsive additives. The printability, resolution, photoresponsiveness and photophysical properties of the polymeric materials demonstrate their potential adaptability and applicability in display technologies and anti‐counterfeiting systems. Here, we described a general pathway for the incorporation of (responsive) luminophores into materials using well‐dispersible and storage‐stable MSNs without losing the luminophore's emission properties, which are not accessible from the sole luminophore due to their low solubility in the 3D printing resin and therefore impede direct incorporation in the resin.

## Results and Discussion

**Figure 1:**
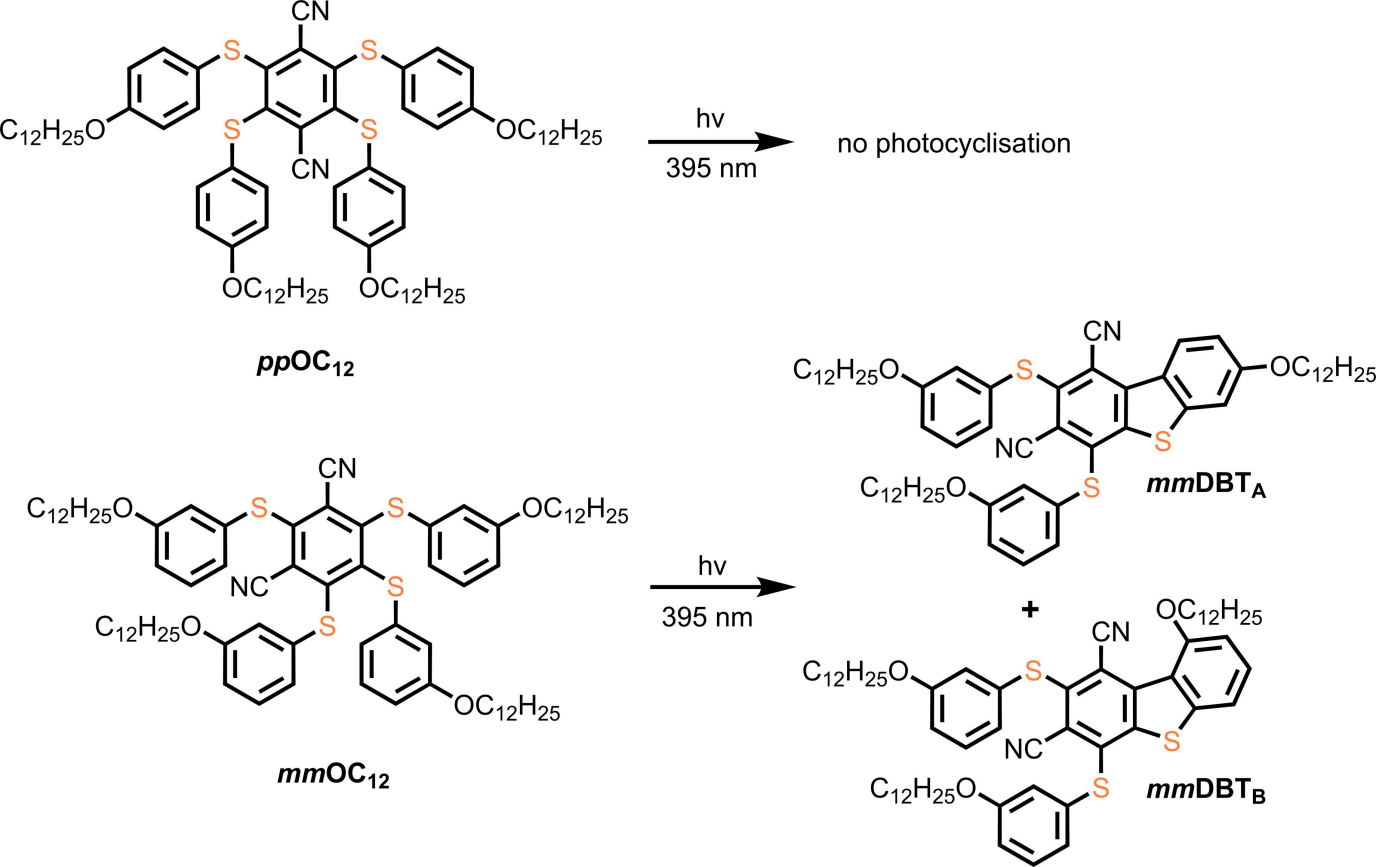
Structures of the two synthesised alkylated tetra‐thioethers (***pp*** **OC_12_** and ***mm*** **OC_12_**) and photoreaction of ***mm*** **OC_12_** to the DBTs ***mm*** **DBT_A_** and ***mm*** **DBT_B._**

The luminophores ***pp*** **OC_12_** and ***mm*** **OC_12_**, which were incorporated in the MSNs, were synthesised from the corresponding tetrachlorodinitriles (tetrachloroterephthalonitrile and tetrachloroisophthalonitrile). The synthesis involved two approaches: i) reaction with a mercaptophenol followed by alkylation, or ii) the formation of an alkylated disulfide, subsequent reduction to the thiol, and reaction under basic conditions with the tetrachlorodinitrile (see Scheme S1, Supporting Information). Both compounds were fully characterised using IR‐ and NMR‐spectroscopy as well as high‐resolution mass spectrometry. The photoreaction of ***mm*** **OC_12_** takes place in solution and polymeric materials upon irradiation with UV‐light (395 nm) and yields the DBTs ***mm*** **DBT_A_** and ***mm*** **DBT_B_** (***mm*** **DBT_A/B_**), which is in good agreement with earlier reports (Figure 1, see mass spectra in Figure S42–45). The hypsochromic shift in the emission spectrum after irradiation (see Figure S37) is likely caused by the increased rigidity of the photoproducts. Due to the ring closure, the molecule can undergo only restricted conformational changes in the excited state, resulting in less energy loss via non‐radiative pathways and a blue‐shifted emission wavelength. The study of previously investigated methoxy‐substituted thioethers suggests that the photocyclisation takes place through the excited triplet state of the molecule.^[42]^

### Characterisation of Luminescent Mesoporous Silica Nanoparticles

**Figure 2:**
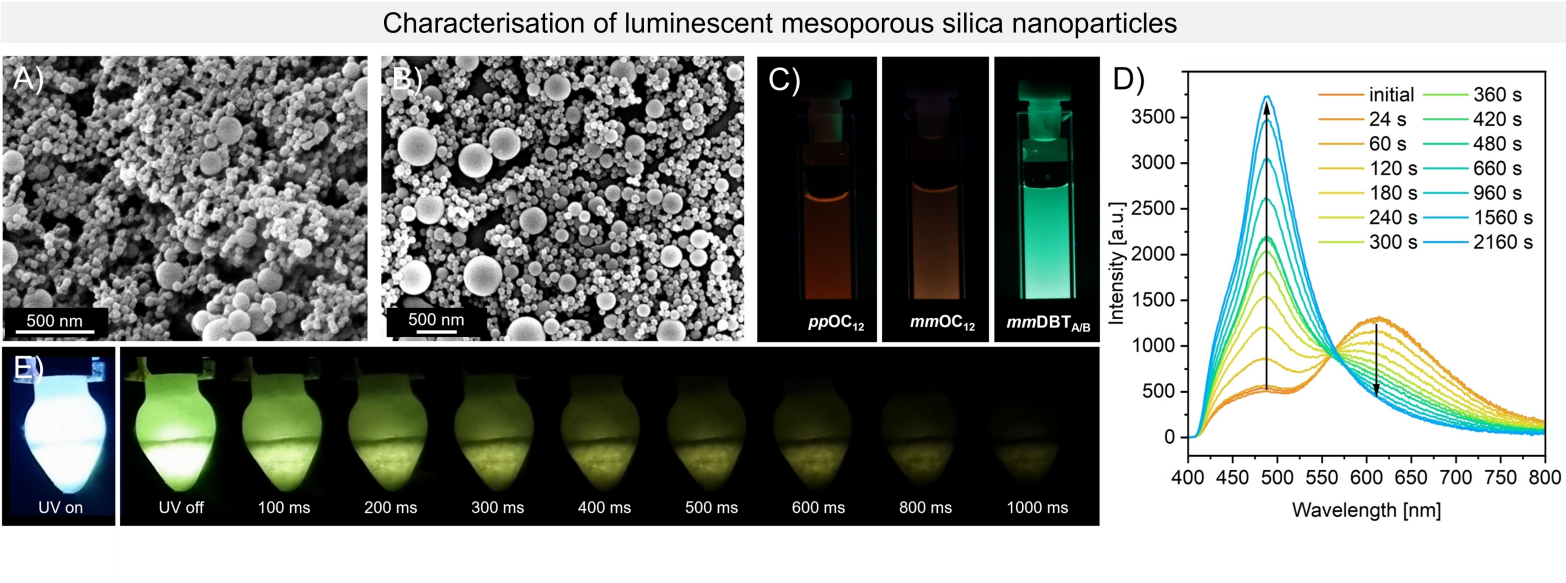
A) SEM image of MSNs with ***pp*** **OC_12_**; B) SEM image of MSNs with ***mm*** **OC_12_**; C) MSNs with ***pp*** **OC_12_**, ***mm*** **OC_12_** and ***mm*** **DBT_A/B_** (obtained after irradiation) in aqueous suspension under UV light (365 nm); D) fluorescence emission spectra of MSN with ***mm*** **OC_12_** at 300 nm excitation after different time of irradiation (also 300 nm) inside the spectrometer as dry MSN powder; E) afterglow of MSNs with ***mm*** **DBT_A/B_** in a frozen methanol matrix at 77 K upon irradiation with UV light (365 nm).

The MSNs were synthesised via the well‐known *Stöber*‐process (*vide infra*). The particles’ ζ‐potential was measured in ultrapure water with a dilute particle suspension (<1 wt.%) in three consecutive DLS (dynamic light scattering) measurements. The average ζ‐potential is shown in the table below. With a ζ‐potential of −24.1 to −38.5 mV, the particles show medium to high stability in aqueous media.^[45]^ The particle size was determined using SEM imaging (Figure 2A/B and S7–9), yielding average diameters between 40 and 90 nm (see Table 1).

**Table 1:** ζ‐potentials and average sizes of the MSNs with and without incorporated luminophore, as well as the mass fraction of luminophore in the MSNs.

Luminophore	ζ‐potential [mV]	Average diameter [nm]	Median diameter [nm]	Mass fraction of luminophore [μg/mg]
No luminophore	−31.3	41.6	41.1	–
***pp*** **OC_12_**	−24.1	53.1	47.7	10.8
***mm*** **OC_12_**	−38.5	88.9	81.3	9.9

The mass fraction of the luminophore in the MSNs was determined using photometry. The mass fraction of ***pp*** **OC_12_** (10.8 μg/mg) is slightly higher than the mass fraction of ***mm*** **OC_12_** (9.9 μg/mg). This difference can be attributed to the better solubility of ***mm*** **OC_12_**, which leads to a loss of luminophore during the washing steps.

Furthermore, the particles’ fluorescence properties were investigated. The MSNs exhibited similar fluorescence in both dry powder and aqueous suspension (see Figure S30/31). The above‐mentioned photoreaction was observed for the ***mm*** **OC_12_** luminophore in both solution and polymer, while no cyclisation was detected for ***pp*** **OC_12_**.[^42^, ^46^] In general, ***mm*** **OC_12_** does not photocyclise in the solid state. However, when ***mm*** **OC_12_** is encapsulated within the MSNs, photocyclisation is observed in both powder (Figure 2D) and aqueous suspension (Figure S38). For MSNs with ***mm*** **OC_12_**, a hypsochromic shift of the emission maximum from 610 nm to 480 nm is observed, indicating the occurrence of a photoreaction. This also aligns with the corresponding CIE plot (Figure S41) and the optical impression (Figure 2C and S22). The observed shift in the emission can be attributed to the different molecular environment present in the MSN system compared to the isolated luminophore in the powder form. In the solid state, the luminophores exist as aggregates or microcrystalline powder which is likely to suppress the photocyclisation. In contrast, within the MSNs, the molecules are probably in a predominantly monomeric state, which avoids aggregation‐based cyclisation hindrance, comparable with the solid powder and hence supports the subsequent formation of dibenzothiophenes (DBTs). In order to confirm that the photoreaction occurs within the particles, the luminophore was extracted after irradiating with UV light (*λ*_exc_=395 nm) using dichloromethane as the solvent. The extracted material was then analysed using high resolution mass spectrometry (Figure S44), which validated the formation of ***mm*** **DBT_A/B_**.

As expected, the ***pp*** **OC_12_** luminophore exhibits no photoreaction under any conditions, whether in the solid state, dissolved, or incorporated within MSNs, which is in good agreement with previous findings (Figure S36).^[46]^ In contrast, MSNs containing ***mm*** **DBT_A/B_** (***mm*** **OC_12_** MSNs after irradiation) displayed a notably long afterglow effect lasting up to 1.4 seconds at 77 K within a frozen matrix (see Figure S16–18), which can be easily observed by the naked eye. A change in the afterglow colour was observed, which is most likely due to the change in the molecular surrounding and, therefore, different interactions with the luminophore. Hence, the amplitude‐weighted average lifetime of the photoluminescence (*τ*_av_) was determined to be 36 ms in the 3D material obtained by stereolithography, which is in good agreement with findings from a previous study.^[42]^

A similar behaviour was observed in PVA (polyvinyl acetate, 87–89 % hydrolysed, 146–186 kDa) films containing MSNs with ***mm*** **DBT_A/B_**. After irradiation, these films exhibited an intense orange afterglow at room temperature, lasting for over 400 ms (see Figure S19).

### Additive Manufacturing Using Luminescent MSNs

**Figure 3:**
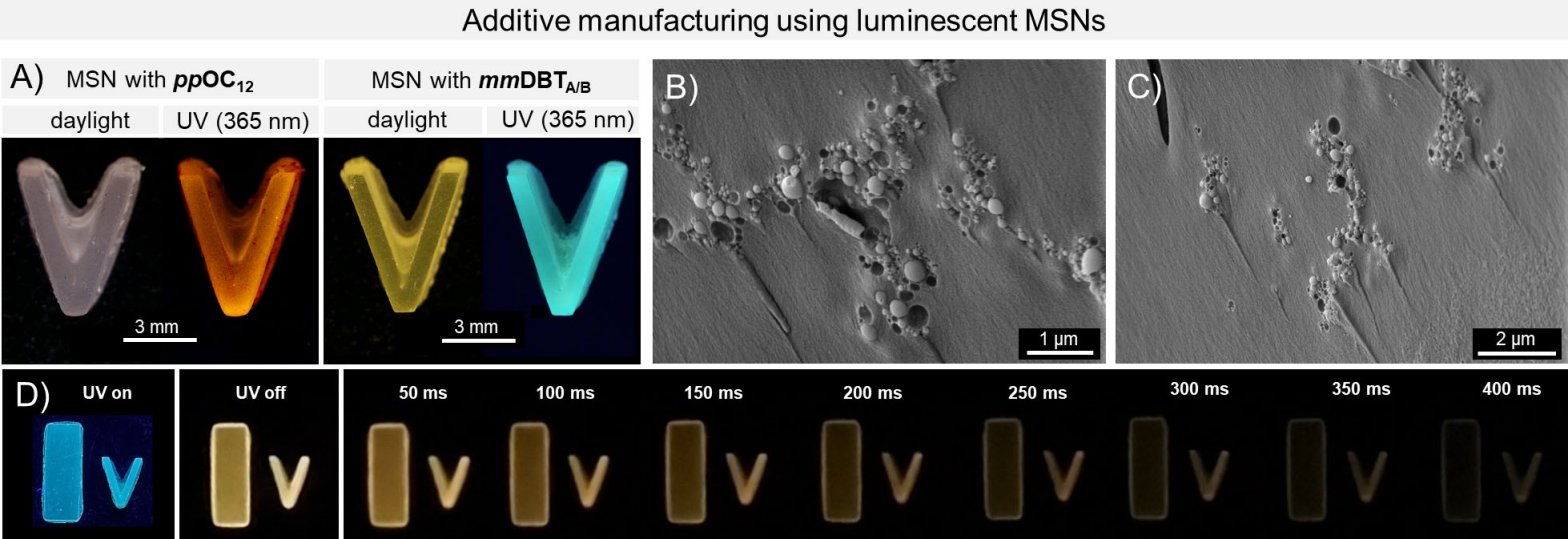
A) 3D‐printed objects with 2 wt.% MSNs under daylight and UV‐light (365 nm); B&C) SEM images of broken edges of 3D‐printed objects with MSNs (***mm*** **OC_12_**) and D) afterglow of 3D‐printed objects with ***mm*** **DBT_A/B_** after irradiation with UV‐light (395 nm, printed strip: 13×5 mm).

The MSNs could be well dispersed in acrylate‐based resins (see experimental section), yielding 3D objects with evenly distributed MSNs. Notably, the MSNs remained in suspension throughout the 3D printing process, preventing the formation of objects with unevenly distributed nanoparticles. A variety of objects with different morphologies were successfully printed. For example, Figure 3D illustrates 3D‐printed V‐shaped objects containing MSNs. SEM images of the broken edge of 3D objects reveal that the MSNs are present as clusters, small groups or individual particles and are evenly distributed (see Figure 3B/C and S10/11). The MSNs help to achieve an evenly distributed colouration throughout the sample, which was not achievable by simply mixing the pure luminophore (especially ***pp*** **OC_12_**) with the resin (confer Figure S23), because of low solubility of the luminophore in the resin.

The 3D‐printed objects containing MSNs exhibit similar luminescent properties as the pure MSNs. During the printing process, partial photoreaction of the ***mm*** **OC_12_** MSNs occurred due to the irradiation of photo‐resin with UV light, which is typically required to initiate the polymerisation of the resin. In contrast, MSNs with ***pp*** **OC_12_** remained non‐photocyclised (see Figure S36). Furthermore, 3D‐printed objects with photocyclised MSNs (***mm*** **DBT_A/B_**) displayed afterglow effects at room temperature, lasting for up to 600 ms after irradiation with UV light (*λ*_exc_ =395 nm, see Figure 3D and S21).

The luminescence lifetimes were determined for the MSN powder and 3D‐printed objects. It was observed that both MSNs with ***mm*** **OC_12_** and ***pp*** **OC_12_** exhibited multiexponential decay. For MSNs with ***mm*** **OC_12_**, a long lifetime (phosphorescence, *τ*_av_=36 ms) was observed in the 3D‐printed objects, while a short component (fluorescence, *τ*_av_=4.85 ns) was observed in the powdered form. However, for MSNs with ***pp*** **OC_12_**, there was no significant difference in lifetimes between the 3D‐printed object and the powder, as it is inherently less emissive (see Table S1). In fact, due to the low emissive nature of MSNs with ***pp*** **OC_12_** in 3D‐printed objects, an extra emission at 460 nm from the photo‐initiator used in the 3D resin (diphenyl(2,4,6‐trimethylbenzoyl)‐phosphine oxide) was observed (Figure S33/34). This was confirmed by measuring the emission lifetime of 3D materials with sole MSNs without luminophores (Figure S28/29).

The difference in lifetimes between the two types of MSNs may be explained by the way these molecules are encapsulated inside the MSNs. In the case of ***mm*** **OC_12_**, it could be encapsulated in a predominantly monomeric form, while ***pp*** **OC_12_** could tend to be encapsulated as an oligomer due to its poor solubility. The oligomeric encapsulation shields ***pp*** **OC_12_** more effectively from triplet dioxygen, resulting in phosphorescence despite not undergoing the photocyclisation to produce the corresponding DBTs. Thus, the packing of these molecules inside the MSNs plays a significant role in the photophysical properties.

In addition, both radiative and non‐radiative rate constants (average *k*_r_ and *k*_nr_, respectively) of the compounds in powder and within the 3D material at room temperature were estimated (see Table S1). Both in powder and in the 3D material, *k*_nr_ vastly surpasses *k*_r_, which indicates strong intermolecular interactions that quench the excited states (an effect also reflected in the low *Φ*_L_). This is illustrated in Figures S24–27 and detailed in Table S1.

### Electrospinning of Nanofibers Containing Luminescent MSNs

To demonstrate the compatibility of luminescent MSNs with polar water‐soluble polymers, PVA was selected as a host material. Initial parameters were selected and further optimised for the present electrospinning device.

All nanofibers (NFs) from parameter optimisation experiments showed narrow diameters of 107–139 nm with small deviations of 14 and 37 nm. Tip‐to‐collector‐distances (TCD) had the greatest influence on NF diameter. As the distance increased, diameters increased starting from 107 nm at 10 cm to 139 nm at 25 cm distance, although the overall change is still small compared to similar studies using different polymers.[^47^, ^48^] A similar trend was observed with varying flow rates, where diameters increased from 116 nm at 1.2 mL/h to 129 nm at 2.4 mL/h. In contrast, increasing voltage reduced diameters, from 127 nm at 20 kV to 107 nm at 28 kV. In general, the morphology of electrospun PVA fibers can shift from uniform to beaded fibers (containing circular cross‐sections) when the composition or electrospinning conditions are altered.^[49]^ Nevertheless, no beads were observed under any parameter configuration, but fiber merging and splitting were evident in all membranes, occurring at varying frequencies. This became the primary criterion for selecting the optimal parameters. Fiber diameters were not measured at merging points to better represent the fibers themselves. They were very uniform aside from the merging and splitting points which is reflected in the low standard deviations of diameters.

**Figure 4:**
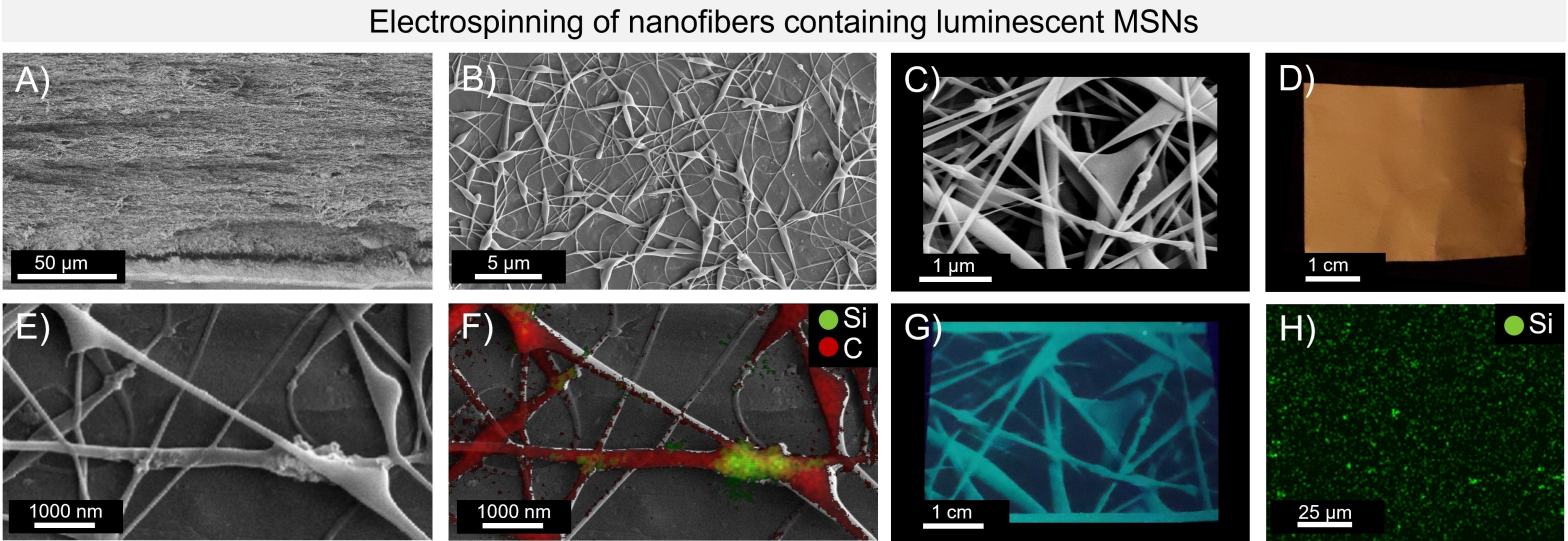
A–C) SEM images of the NFs with 6 wt.% ***mm*** **OC_12_** MSNs with different magnifications; D) photo of electrospun NFs on aluminium foil; E/F) SEM image of the NF with ***mm*** **OC_12_** MSNs; (E) the same image with spatially resolved EDX measurements for the elements silicon (green) and carbon (red) representing the presence of polymer and MSNs; G) image of the NFs inscribed into the electrospun fleece using a photomask of the SEM image; H) spatially resolved EDX surface analysis of the NFs with ***mm*** **OC_12_** MSNs showing the presence of silica throughout the NF sample.

MSNs appeared to form a few larger clusters when embedded in the membranes. However, many smaller groups were visibly enclosed within the NFs, as confirmed by EDX surface analysis (Figure 4F and S13/14). In addition, EDX surface analysis demonstrated that the MSNs were evenly distributed throughout the NF sample. (Figure 4H and S15). After incorporating the ***mm*** **OC_12_** MSNs inside the NFs, a partial photoreaction had already occurred, as indicated by the increased intensity of the single band at 490 nm (Figure S40). This could be attributed to irradiation exposure within the electrospinning apparatus during the spinning process or to electronically induced cyclisation.^[50]^ The corresponding photoproducts, ***mm*** **DBT_A/B_**, were extracted from the irradiated NFs containing ***mm*** **OC_12_** MSNs with DCM and subsequently identified via high‐resolution mass spectrometry (Figure S45).

The spun material demonstrates interesting properties that enable to inscribe patterns or symbols. After partial irradiation with UV light (*λ*_exc_=395 nm) using a photomask (in this case a SEM image), the photoreaction was initiated in the irradiated areas, causing these regions to appear brighter (green‐blueish) compared to the non‐irradiated sections. This property allows the possibility of inscribing symbols, texts and even images into the material, which are invisible under daylight, but become visible under UV light (see Figure 4G and Scheme S3). Additionally, an afterglow effect was observed at 77 K for the NFs irradiated with UV‐light, with an average duration of 1370 ms (see Figure S20) as detectable by bare eyes.

## Experimental Section

### Synthesis of Luminescent Mesoporous Silica Nanoparticles

The MSNs were synthesised in a *Stöber*‐like process (see Scheme S2). Initially, 80 mg cetyltrimethylammonium bromide (CTAB) and 100 mg tris(hydroxymethyl)amino‐methane (TRISMA) were dissolved in 15 mL of water. Subsequently, 5 mg of the luminophore was dissolved in 2 mL of ethanol (***mm*** **OC_12_**) or THF (***pp*** **OC_12_**) and added to the CTAB/TRISMA solution, which was further stirred for 15 minutes. Following this, 700 μL of tetraethyl orthosilicate (TEOS) was introduced to the mixture with vigorous stirring (750 rpm). The reaction mixture was allowed to stir overnight, then centrifuged and washed several times with copious amounts of water, followed by a wash with ethanol. The resulting material was dried under vacuum or lyophilised, yielding a fine powder. The obtained nanoparticle powder can be readily redispersed in water or alcohol using ultrasound.

### Preparation of the 3D Printing Resin

The 3D printing resin was prepared by mixing 60 wt.% pentaerythritol tetraacrylate as crosslinker, 30 wt.% poly(ethylene glycol) methyl ether methacrylate as monomer, 9 wt.% 2‐[[(butylamino)carbonyl]oxy]ethyl acrylate as diluent and 1 wt.% diphenyl(2,4,6‐trimethylbenzoyl)phosphine oxide as photoinitiator. Prior to printing of the objects with MSNs, 2 wt.% MSNs were thoroughly suspended in the resin using an ultrasonic bath and magnetic stirring.

### Stereolithographic Printing (SLA)

3D printing was performed using an Anycubic Photon Mono 4 K DLP (digital light processing) printer with a 405 nm LED light source with an irradiance of 4.2 mW/cm^2^. The print models were constructed using Inventor Professional Version 2024.2 from Autodesk and exported as STL files. After that, all samples were sliced with Anycubic Photon Workshop (Version v3.2.2) and printed under the same conditions of layer thickness 0.1 mm, normal exposure time 14.0 s, off time 0.5 s, bottom exposure time 14.0 s, bottom layers 6, anti‐alias 1, z lift distance 6 mm, z lift speed 4 mm/s and z retract speed 6 mm/s. Typically, 4 g of resin with 80 mg of suspended nanoparticles (2 wt.%) were added into a custom resin tub with a reduced volume. After printing, the samples were washed with isopropanol to remove any unpolymerised resin.

### Electrospinning of Nanofibers with MSNs

Electrospinning was carried out in a horizontal arrangement with temperatures of 20–22 °C and a humidity of 50 %. The polymer solution was poured into a syringe (5 mL, 20 G) and fixated. For parameter optimisation tip‐to‐collector distances (TCDs) of 10 to 25 cm, voltages between 20 and 30 kV, and feed rates of 1.5 to 2.4 mL/h were used while the drum collector was not rotating (0 rpm). The best parameters were identified with SEM imaging and used for creating membranes from MSN containing solutions (TCD: 15 cm, 28 kV, 1.8 mL/h). For the size increase of these membranes, the metal drum collector was rotated at 315 rpm. Electrospun membranes were analysed using SEM imaging. Diameters of nanofibers (NFs) were measured using Fiji software (v.2.15.1).

## Conclusions

In conclusion, in this study we demonstrate mesoporous silica nanoparticles (MSNs) as efficient carriers for integrating nonpolar and poorly soluble organic luminophores into aqueous media and other polar environments. MSNs facilitate the stable dispersal of synthesised luminophores (***mm*** **OC_12_** and ***pp*** **OC_12_**) into materials like PVA films, 3D‐printed resins, and electrospun nanofibers, preserving their photophysical properties, which are typically prone to quenching or degradation. This encapsulation process allows luminophores to undergo solvent‐free photoreactions, notably the light‐induced cyclisation of ***mm*** **OC_12_** to form dibenzothiophenes (DBTs) with long‐lasting afterglow when exposed to UV light. The study also highlights the compatibility of MSNs with SLA printing and electrospinning. These techniques allow for the creation of intricate designs and nanofibers embedded with evenly distributed luminophores, maintaining their light‐responsive behaviour.

As MSNs are stable, easy to handle and can be stored on the long‐term as powders, they are suitable for scalable applications. Their non‐air‐sensitive nature, along with compatibility with polar and aqueous media, highlights their potential contribution towards environmentally friendlier processes. This approach opens new paths for biomedical applications, wastewater treatment, and functional nanomaterials that generate reactive oxygen species, particularly if porous structures are attained that allow for diffusion of dioxygen while containing molecular species that sustain long‐lived photoexcited states (micro‐ or milliseconds and above). Overall, the combination of luminophore‐embedded MSNs with advanced manufacturing techniques paves the way for useful applications like advanced displays and anti‐counterfeiting systems, where extended luminescence is highly advantageous.

## Supporting Information Summary

The authors have cited additional references within the Supporting Information.^[51]^

## Conflict of Interests

The authors declare no conflict of interest.

1

## Supporting information

As a service to our authors and readers, this journal provides supporting information supplied by the authors. Such materials are peer reviewed and may be re‐organized for online delivery, but are not copy‐edited or typeset. Technical support issues arising from supporting information (other than missing files) should be addressed to the authors.

Supporting Information

## Data Availability

The data that support the findings of this study are available in the supplementary material of this article.
