# Supplementary material for: Photoresponsive Luminescent Silica Nanoparticles as Additive for 3D Printing and Electrospinning
Source: Chem Asian J. 2024 Dec 12;20(4):e202401415. doi: 10.1002/asia.202401415 (PMC11826982; doi:10.1002/asia.202401415)
Supplement: Supplementary file 1 — Supporting Information [file ASIA-20-e202401415-s001.pdf]

Supporting Information

Photoresponsive Luminescent Silica Nanoparticles as Additive for 3D Printing and Electrospinning

Rick Y. Lorberg, Sidharth Thulaseedharan Nair Sailaja, Fabian Terlau, Maria Victoria Cappellari, Marco Schmiedtchen, Anzhela Galstyan, Cristian A. Strassert, Michael Giese and Jens Voskuhl*

Table of contents

General Methods **3**
Experimental Section **5**
2,3,5,6-Tetrakis((4-hydroxyphenyl)sulphanyl)terephthalonitrile (*pp*OH) 6
2,3,5,6-Tetrakis((4-(dodecyloxy)phenyl)sulphanyl)terephthalonitrile (*pp*OC_{12}) 6
Bis-(3-dodecyloxyphenyl)-disulphide (5) 7
2,4,5,6-tetrakis((3-(dodecyloxy)phenyl)sulphanyl)isophthalonitrile (*mm*OC_{12}) 8
NMR Spectra **9**
General Procedures **12**
MSNs with Luminophore 12
MSNs without Luminophore (*Stöber* process) 13
Thin Films 13
Electrospinning 13
Inscribing in NFs 13
SEM Imaging **14**
MSNs 14
MSNs in 3D Material 16
MSNs in PVA Nanofibers 18
Afterglow Experiments **19**
Photographs of MSN Powders and 3D objects **22**
Photophysical Characterisation **23**
Lifetimes and Quantum yields 23
Fluorescence Spectra 27
Irradiation Experiments 30
Mass Spectra **33**
References **37**

General Methods

Chemicals

Chemicals were used without further purification. Chemicals were purchased from following vendors: Carl Roth (Karlsruhe, Germany), Merck (Darmstadt, Germany), TCI (Tokyo, Japan), Fisher Scientific (Hampton, New Hampshire, United States) and Deutero (Kastellaun, Germany). Potassium carbonate was stored at 90 °C prior to use. The nanoparticle synthesis was carried out in ultrapure water from a BerryPURE® mini ultrapure water system, where conductivity was less than 0.055 µS/cm.

Chromatography

Polygram SIL G/UV254 (MACHEREY-NAGEL, Düren, Germany) silica plates were used for thin-layer chromatography (TLC). Column chromatography was performed using silica gel with particle size 40-63 µM (MACHEREY-NAGEL, Düren, Germany) and only distilled or p.a. grade solvents were used.

Mass spectrometry

High resolution mass spectrometry was performed on a Bruker maXis 4G Q-TOF-mass spectrometer (Billerica, Massachusetts, United States), using electro spray or atmospheric pressure chemical ionisation (ESI/APCI) also from Bruker.

Scanning electron microscopy (SEM)

Scanning electron microscopy images of the MSNs and fibers were taken with an Apreo S LoVac microscope from Thermo Fisher Scientific (Waltham, Massachusetts, United States) and were sputtered with gold and palladium. SEM images of the 3D objects with MSN were taken on a Crossbeam 540 microscope from ZEISS (Oberkochen, Germany).

Additive manufacturing

A Photon Mono 4K 3D-printer from Anycubic (Shangjing, China) was used for the of printing the 3D-Objects. The used polymer resin was a four-component mixture of 1 wt.% diphenyl(2,4,6-trimethylbenzoyl)phosphinoxide as photoinitiator, 60 wt.% pentaerythrit-tetraacrylate as crosslinker, 30 wt.% poly-(ethylenglykol)-methylether-methacrylate as monomer and 9 wt.% acrylicacid-{2-(((butylamino)-carbonyl)-oxy)-ethylester} as diluent.

NMR spectroscopy

NMR spectra were obtained with an AVANCE NEO400 (400 MHz ^{1}H, 101 MHz ^{13}C) spectrometer from Bruker (Billerica, Massachusetts, United States) at 298 K. ^{13}C spectra were recorded with proton decoupling. Either acetone-d_6, dichloromethane-d_2 or deuterated chloroform was used as solvent. The signal of the residual protium species of the solvents were used for referencing the spectra (aceton-d_6: ^{1}H: δ = 2.05 ppm, ^{13}C: 29.84, 206.26 ppm, dichloromethane-d_2: ^{1}H: δ = 5.32 ppm, ^{13}C: 53.84 ppm and $CDCl_3$: ^{1}H: δ = 7.26 ppm, ^{13}C: 77.16 ppm). The signals were assigned to the corresponding nuclei by 2D-spectra (HSQC-, HMBC- and COSY-spectra). They are labelled to the corresponding number of the nuclei in the shown structural formula (Cq stands for any quaternary carbon nuclei, unless the assigned ones). Chemical shifts δ are given in ppm (parts per million) and coupling constants J in Hz (Hertz). Multiplets are reported with the following abbreviations: s (singlet), d (doublet), t (triplet), pseudo-triplet (pst), q (quartet) and m (multiplet).

Dynamic light scattering (DLS)

Dynamic light scattering and ζ-potential measurements were performed with a Zetasizer Nano-ZS from Malvern Pananalytic (Malvern, United Kingdom).

Fluorescence Spectroscopy

Excitation and emission spectra were obtained with an RF-6000 spectrometer from Shimadzu (Kyoto, Japan). Quartz cuvettes from Hellma (Müllheim im Marktgräflerland, Germany) with a thickness of 10 x 4 mm were used for measurements in aqueous suspension. Solid probes were measured between two quartz glass slides from Thermo Fisher Scientific (Waltham, Massachusetts, United States). The lamp signal was filtered with a 420 or 400 nm long pass filter from Shimadzu (Kyoto, Japan). The scattered light signals in the emission spectra were filtered by using a 320 nm short pass filter from the same company.

Steady-state emission spectra were recorded on a FluoTime 300 spectrometer from PicoQuant (Berlin, Germany) equipped with a 300 W ozone-free Xe lamp (200-1100 nm), a 10 W Xe flash-lamp (200-1100 nm, pulse width ~ 1 µs) with repetition rates of 1 – 300 Hz, a double-grating excitation monochromator (Czerny-Turner type, grating with 1200 lines/mm, blaze wavelength: 300 nm), diode lasers (pulse width < 20 ps) operated by a computer-controlled laser driver PDL-820 "Sepia II" (repetition rate up to 80 MHz, burst mode for slow and weak decays), two emission monochromators (Czerny-Turner, selectable between double-grating blazed at 500 nm with 2.7 nm/mm dispersion and 1200 lines/mm, or single-grating blazed at 1250 nm with 5.4 nm/mm dispersion and 600 lines/mm) with adjustable slit width between 25 µm and 7 mm, Glan-Thompson polarisers for excitation (after the Xe-lamps) and emission (after the sample). Different sample holders (Peltier-cooled mounting unit ranging from -15 to 110 °C or an adjustable front-face sample holder), along with two detectors (namely a PMA Hybrid-07 from PicoQuant with transit time spread FWHM < 50 ps, 220 – 850 nm, or a H10330C-45-C3 NIR detector with transit time spread FWHM 0.4 ns, 950-1400 nm from Hamamatsu Photonics, Ltd., Shizuoka, Japan) were used. Steady-state spectra and photoluminescence lifetimes were recorded in TCSPC mode by a PicoHarp 300 (minimum base resolution = 4 ps) or in MCS mode by a TimeHarp 260 (where up to several ms can be traced). Emission spectra were corrected for source intensity (lamp and grating) by standard correction curves. For samples with lifetimes in the ns order, an instrument response function calibration (IRF) was performed using a diluted Ludox® dispersion. Lifetime analysis was performed using the commercial EasyTau 2 software (PicoQuant). The quality of the fit was assessed by minimizing the reduced chi-squared function (χ^2) and visual inspection of the weighted residuals and their autocorrelation. All solvents used were of spectrometric grade (Uvasol®, Merck).

Photoluminescence quantum yields were measured with a Hamamatsu Photonics absolute PL quantum yield measurement system (C9920-02) equipped with an L9799-01 CW Xe light source (150 W), a monochromator, a C7473 photonic multi-channel analyser, an integrating sphere and employing U6039-05 software (Hamamatsu Photonics Ltd.).

Experimental Section

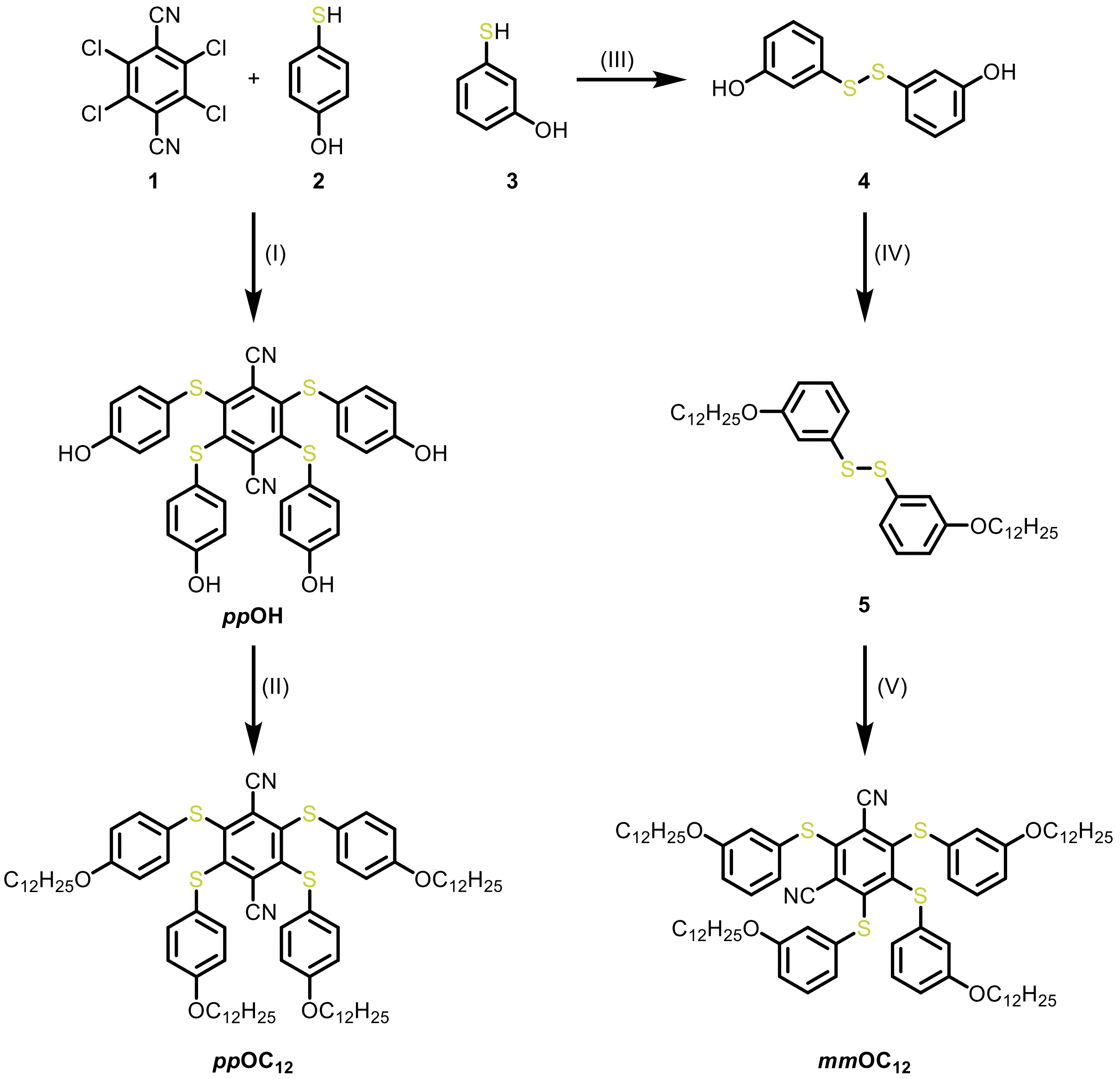

Scheme S1: Reaction scheme to the two alkylated luminophores ***pp*OC$_{12}$** and ***mm*OC$_{12}$**. (I) K_2CO_3 (18 eq.), DMF, 50 °C, 18 h; (II) $BrC_{12}H_{25}$ (8 eq.), K_2CO_3 (12 eq.), DMF, 50 °C, 64 h; (III) air, K_2CO_3 (2 eq.), ACN, rt, 2 h; (IV) $BrC_{12}H_{25}$ (1.1 eq.), ACN, 82 °C, 14 h; (V_A) $NaBH_4$ (5 eq.), DMF, 100 °C, 2 h; (V_B) tetrachloroisophthalonitrile (0.17 eq.), K_2CO_3 (12 eq.), DMF, 50 °C, 14 h.

2,3,5,6-Tetrakis((4-hydroxyphenyl)sulphanyl)terephthalonitrile (*pp*OH)

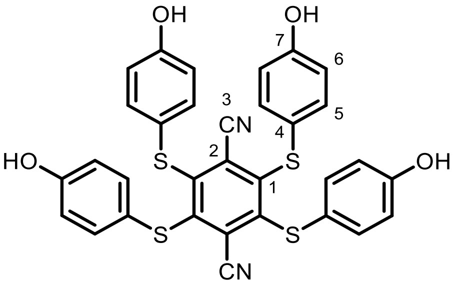

***pp*OH**

***pp*OH** was synthesised according to a literature procedure.[1] 2,3,5,6-Tetrachloroterephthalonitrile (522.8 mg, 1.966 mmol, 1 eq.), 4-hydroxythiophenol (1.490 g, 11.809 mmol, 6 eq.) and potassium carbonate (5.071 g, 36.691 mmol, 18 eq.) were suspended in 16 mL dry DMF and heated overnight at 50 °C in a closed reaction tube. After completion of the reaction, the mixture was given on 50 mL half-concentrated ice-cooled hydrochloric acid. The crude product was filtered off and washed with cyclohexane. The product was purified *via* recrystallisation from acetone and dried *in vacuo*.

Yield: 875.0 mg (1.401 mmol, 71.4 %), orange solid.

M ($C_{32}H_{20}N_2O_4S_4$): 624.77 g/mol.

^{1}H-NMR (400 MHz, acetone-*d*$_6$): δ = 8.32 (s, 4H, H-OH), 7.21 – 7.14 (m, 8H, H-5), 6.80 – 6.76 (m, 8H, H-4).

^{13}C-NMR (101 MHz, acetone-*d*$_6$): δ = 158.65 (C7), 147.60 (C1), 134.01 (C6), 128.90 (C2), 124.37 (C4), 117.40 (C5), 115.40 (C3).

The analytical data match the ones reported.[1]

2,3,5,6-Tetrakis((4-(dodecyloxy)phenyl)sulphanyl)terephthalonitrile (*pp*OC$_{12}$)

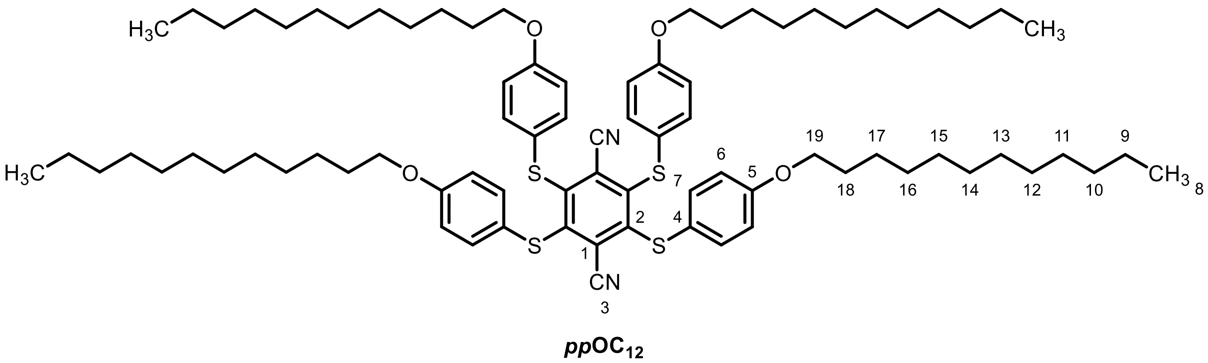

***pp*OC$_{12}$**

2,3,5,6-Tetrakis((4-hydroxyphenyl)sulphanyl)terephthalonitrile (112.8 mg, 0.181 mmol, 1 eq.), 1-bromododecane (424 µL, 1.767 mmol, 10 eq.) and potassium carbonate (371.1 mg, 2.685 mmol, 15 eq.) were suspended in 10 mL dry DMF and heated for three days at 50 °C in a closed reaction tube. After completion of the reaction, the mixture was given on 50 mL distilled water. The mixture was extracted with ethyl acetate and dried over magnesium sulphate. After drying, the drying agent was filtered off, and the solvent was removed *in vacuo*.

The crude product was purified via column chromatography on silica gel (eluent: cyclohexane : DCM = 7 : 3 → 6 : 4).

Yield: 165.0 mg (0.127 mmol, 70.5 %), red solid.

M ($C_{80}H_{116}N_2O_4S_4$): 1298.06 g/mol.

Melting Point: 136-137 °C

^{1}H-NMR (400 MHz, CD_2Cl_2): δ = 7.19 – 7.13 (m, 8H, H-6), 6.80 – 6.74 (m, 8H, H-7), 3.92 (t, J = 6.6 Hz, 8H, H-19), 1.80 – 1.71 (m, 8H, H-18), 1.48 – 1.38 (m, 8H, H-17), 1.34 – 1.17 (m, 64H, H-9–16), 0.88 (t, J = 6.9 Hz, 12H, H-8).

^{13}C-NMR (101 MHz, CD_2Cl_2): δ = 160.07 (Cq), 147.42 (Cq), 133.89 (C6), 128.12 (Cq), 124.81 (Cq), 116.00 (C7), 115.15 (C3), 68.81 (C19), 32.49 (C9-16), 30.24 (C9-16), 30.21 (C9-16), 30.18 (C9-16), 30.16 (C9-16), 29.99 (C9-16), 29.92 (C9-16), 29.77 (C18), 26.56 (C17), 23.25 (C9-16), 14.44 (C8).

IR: $\tilde{\nu}$ = 2953 (w), 2916 (s), 2871 (w), 2848 (s), 1595 (w), 1574 (w), 1495 (s), 1468 (m), 1390 (w), 1380 (w), 1308 (w), 1286 (w), 1250 (s), 1225 (s), 1178 (m), 1154 (w), 1109 (w), 1089 (w), 1073 (w), 1035 (w), 1005 (w), 958 (w), 916 (w), 867 (w), 814 (s), 753 (w), 720 (w), 664 (w), 650 (w), 611 (w), 582 (w), 514 (w), 486 (w), 472 (w), 463 (w), 443 (w), 434 (w), 419 (w), 411 (w) cm^{-1}.

HR-MS (APCI-pos.), *m/z*: Calculated: $[M+H]^+$: 1297.7891, found: 1297.7886.

Bis-(3-dodecyloxyphenyl)-disulphide (5)

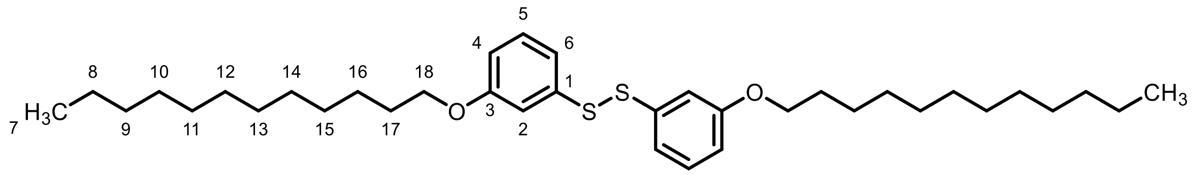

5

3-Hydroxythiophenol (500 µL, 4.902 mmol, 1 eq.) was dissolved in 20 mL acetonitrile. Then potassium carbonate (1.525 g, 11.034 mmol, 2.3 eq.) was added and suspended in the solution. The mixture was stirred in an open beaker overnight. The completion of the reaction was checked via thin layer chromatography and NMR spectroscopy. After that, 1-bromododecane (1.300 mL, 5.425 mmol, 1.1 eq.) was added to the suspension, and the reaction mixture was boiled under reflux at 82 °C overnight. The carbonate was filtered off and washed with ethyl acetate. The solvent was distilled off under reduced pressure and the alkylated disulphide was purified *via* column chromatography (eluent: cyclohexane : ethyl acetate = 100 : 0 → 100 : 5).

Yield: 1.44 g (quantitative), colourless solid.

M ($C_{36}H_{58}O_2S_2$): 586.97 g/mol.

Melting Point: 48 °C

^{1}H-NMR (400 MHz, Acetone-*d*$_6$): δ = 7.29 – 7.24 (m, 2H, H-5), 7.10 – 7.07 (m, 4H, H-4, H-6), 6.85 – 6.81 (m, 2H, H-2), 3.96 (t, J = 6.5 Hz, 4H, H-18), 1.77 – 1.69 (m, 4H, H-17), 1.49 – 1.40 (m, 4H, H-16), 1.37 – 1.27 (m, 32H, H-8–15), 0.88 (t, J = 6.8 Hz, 6H, H-7).

^{13}C-NMR (101 MHz, Acetone-d_6): δ = 160.75 (Cq), 138.63 (Cq), 131.05 (C5), 119.98 (C4/C6), 114.67 (C2), 113.76 (C4/C6), 68.72 (C18), 32.65 (C8-15), 30.39 (C8-15/C17), 30.37 (C8-15/C17), 30.33 (C8-15/C17), 30.32 (C8-15/C17), 30.08 (C8-15/C17), 26.70 (C16), 23.33 (C8-15), 14.37 (C7).

IR: $\tilde{\nu}$ = 2952 (w), 2915 (s), 2846 (w), 2746 (s), 1588 (w), 1572 (w), 1475 (s), 1465 (m), 1446 (w), 1429 (w), 1386 (w), 1283 (w), 1228 (s), 1174 (s), 1146 (m), 1129 (w), 1095 (w), 1073 (w), 1044 (w), 1033 (w), 1016 (w), 1000 (w), 961 (w), 920 (w), 889 (s), 867 (w), 856 (w), 841 (w), 803 (w), 768 (w), 718 (w), 690 (w), 680 (w), 613 (w), 590 (w), 580 (w), 563 (w), 534 (w), 526 (w), 514 (w), 494 (w), 473 (w), 468 (w), 455 (w), 441 (w) cm^{-1}.

HR-MS (ESI-pos.), *m/z*: Calculated: [M+H]$^+$: 587.3951, found: 587.3942; Calculated: [M+Na]$^+$: 609.3770, found: 609.3760, Calculated: [M+K]$^+$: 625.3510, found: 625.3499.

2,4,5,6-tetrakis((3-(dodecyloxy)phenyl)sulphanyl)isophthalonitrile (*mm*OC$_{12}$)

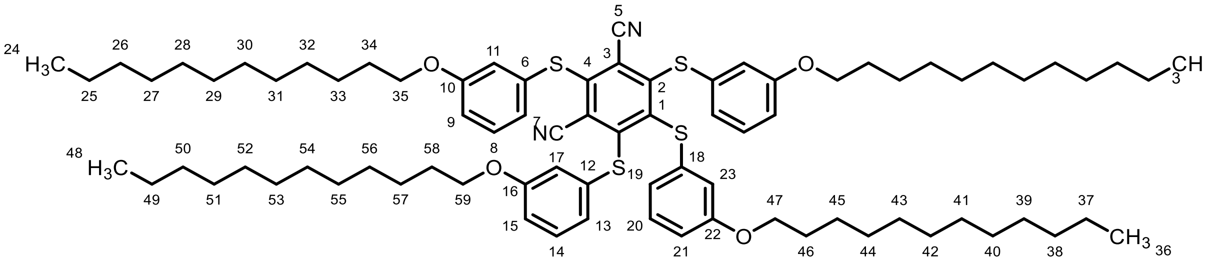

***mm*OC$_{12}$**

Bis-(3-dodecyloxyphenyl)-disulphide (**5**) (596.8 mg, 1.017 mmol, 1 eq.) was suspended in 7 mL of dry DMF. After the addition of sodium borohydride (194.3 mg, 5.136 mmol, 5 eq.), the reaction was heated for two hours at 100 °C. After that, the reaction was cooled with an ice bath and stopped by the addition of hydrochloric acid, bringing the pH down to 2. The quenched reaction mixture was extracted with ethyl acetate, the organic phase was dried over magnesium sulphate, and the solvent was distilled off under reduced pressure. The resulting 3-dodecyloxythiophenol was used without further purification.

Yield: 675.7 mg (quantitative), colourless solid.

HR-MS (ESI-pos.), m/z: Calculated: [M+H]$^+$: 295.2090, found: 295.2091.

2,4,5,6-Tetrachlorisophthalonitrile (67.4 mg, 0.253 mmol, 1 eq.), potassium carbonate (520.9 mg, 3.769 mmol, 12 eq.) and 3-dodecyloxythiolphenol (441.8 mg, 1.500 mmol, 6 eq.) were suspended in 8 mL dry DMF and heated protected from light overnight at 50 °C. After completion of the reaction, the mixture was given on 50 mL distilled water. The mixture was extracted with ethyl acetate and dried over magnesium sulphate. After drying, the drying agent was filtered off, and the solvent was removed *in vacuo*. The crude product was purified via column chromatography on silica gel (eluent: *n*-hexane : DCM = 7 : 3 → 7 : 4). In a second step the not yet clean mixture was purified using a NEXTGEN automated flash chromatography (eluent: n-hex : ethyl acetate = 100 : 1 → 100 : 5).

Yield: 223.6 mg (68.0 %), yellow resin.

M ($C_{80}H_{116}N_2O_4S_4$): 1298.06 g/mol.

^{1}H-NMR (400 MHz, CDCl$_3$): δ = 7.16 – 7.10 (m, 4H, H-8, H-14, H-20), 6.93 (pst, J = 2.1, 1.98 Hz, 1H, Ar-H), 6.83 – 6.70 (m, 7H, Ar-H), 6.70 – 6.66 (m, 2H, Ar-H), 6.61 (pst, J = 1.95,

2.15 Hz, 1H, Ar-H), 6.56 – 6.52 (m, 1H, Ar-H), 3.90 – 3.81 (m, 8H, H-35, H-47, H-59), 1.78 – 1.69 (m, 8H, H-34, H-46, H-58), 1.45 – 1.38 (m, 8H, H-33, H-45, H-57), 1.36 – 1.21 (m, 64H, H-25–32, H-44–37, H49–56), 0.88 (t, J = 6.8 Hz, 12H, H-24, H-36, H-48).

^{13}C-NMR (101 MHz, $CDCl_3$): δ = 159.91 (C-Ar), 159.81 (C-Ar), 151.79 (Cq), 145.83 (Cq), 136.57 (C8/C14/C20), 135.17 (C8/C14/C20), 133.74 (C8/C14/C20), 130.39 (Cq), 130.34 (Cq), 124.21 (Cq), 123.62 (C-Ar), 122.84 (C-Ar), 120.60 (2C, C-Ar), 117.70 (C-Ar), 117.03 (2C, C-Ar), 115.42 (C-Ar), 114.87 (C-Ar), 114.78 (2C, C-Ar), 114.06 (Cq), 113.58 (Cq), 68.31 (C34/C46/C58), 32.07 (C24/C36/C48), 29.83 (CH_2), 29.79 (CH_2), 29.77 (CH_2), 29.74 (CH_2), 29.59 (CH_2), 29.51 (CH_2), 29.33 (C35/C47/C59), 26.16 (C35/C47/C59), 22.84 (C24/C36/C48), 14.27 (CH_2), mit C-Ar = C7/C9/C11/C13/C15/C17/C19/C21/C23 und CH_2 = C25-34/C37-C46/C49-58.

IR: $\tilde{\nu}$ = 2918 (s), 2850 (s), 2489 (w), 2228 (w), 1587 (s), 1498 (w), 1481 (m), 1468 (s), 1421 (w), 1391 (w), 1330 (w), 1312 (w), 1284 (w), 1245 (s), 1160 (w), 1126 (w), 1098 (w), 1071 (w), 1023 (m), 992 (w), 969 (w), 925 (w), 880 (w), 836 (w), 776 (m), 720 (w), 682 (m), 601 (w), 577 (w), 545 (w), 520 (w), 512 (w), 499 (w), 483 (w), 468 (w), 443 (w), 434 (w), 425 (w), 418 (w), 406 (w) cm^{-1}.

HR-MS (ESI-pos.), *m/z*: Calculated: $[M+H]^+$: 1297.7891, found: 1297.7885; Calculated: $[M+Na]^+$: 1319.7710, found: 1319.7703, Calculated: $[M+K]^+$: 1335.7450, found: 1335.7450.

NMR Spectra

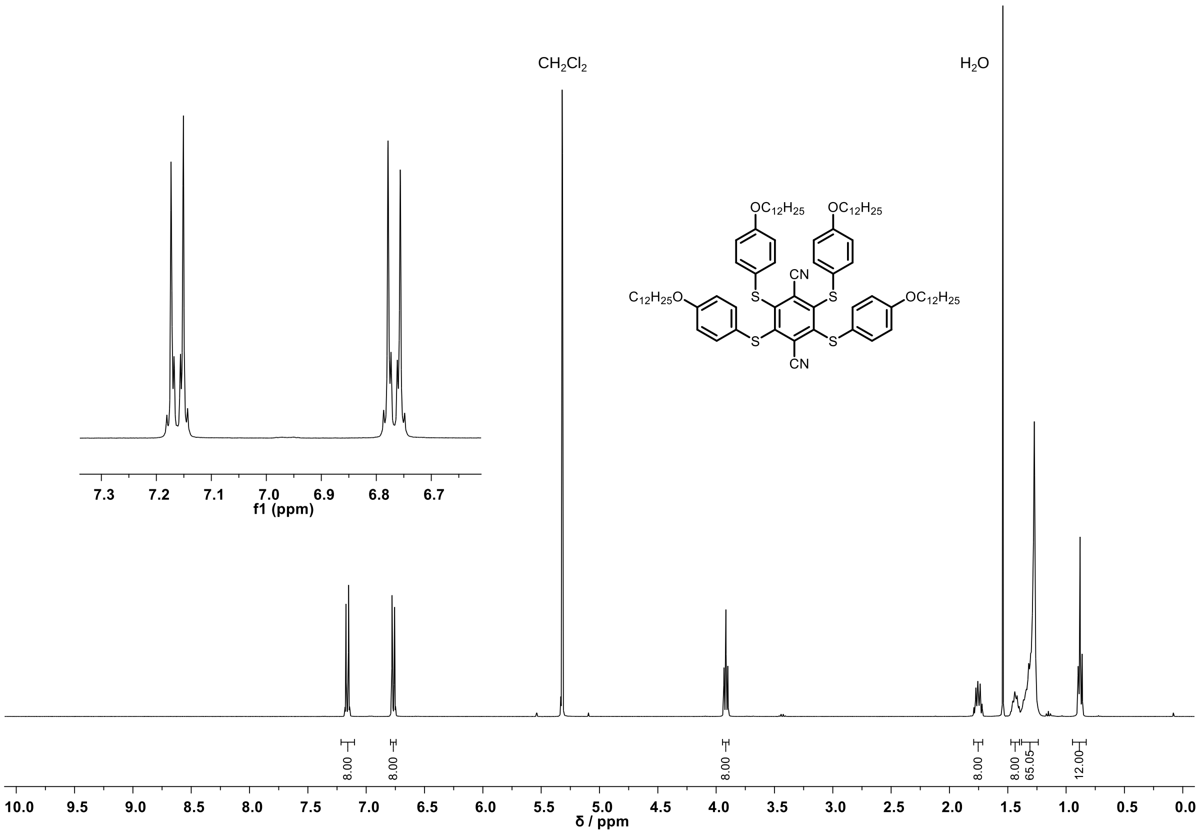

Figure S1: ^{1}H-NMR spectrum (400 MHz, 298 K, dichloromethane-d_2) of ***pp*OC$_{12}$**.

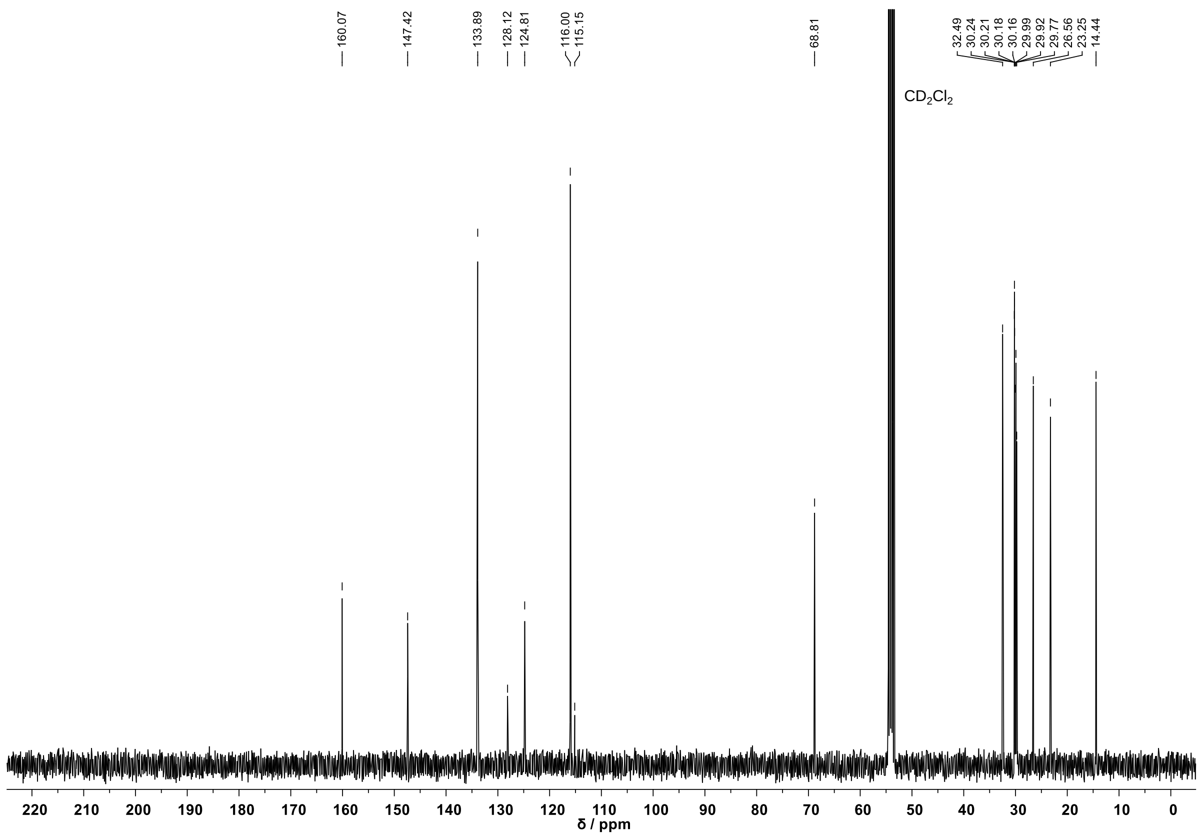

Figure S2: ^{13}C- NMR spectrum (101 MHz, 298 K, dichloromethane-d_2) of ***pp*OC$_{12}$**.

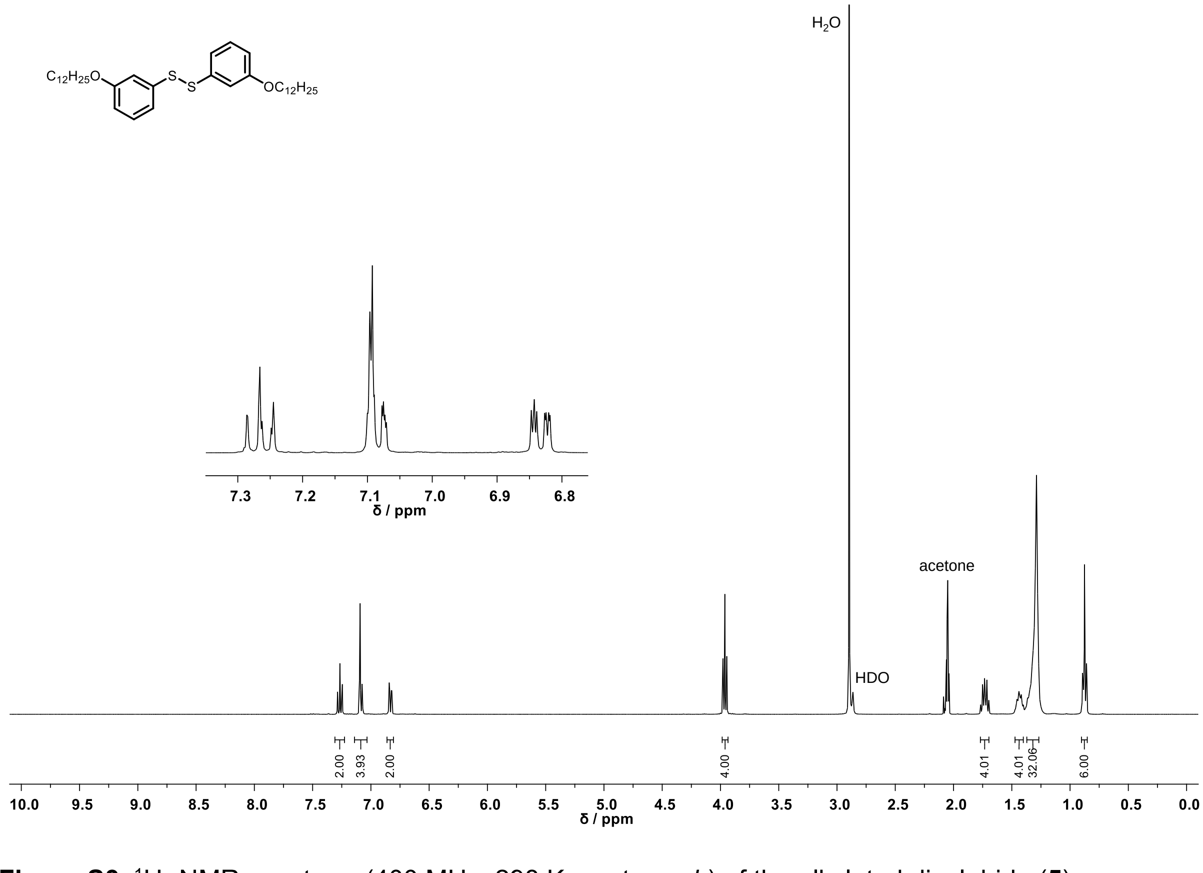

Figure S3: ^{1}H- NMR spectrum (400 MHz, 298 K, acetone-d_6) of the alkylated disulphide (**5**).

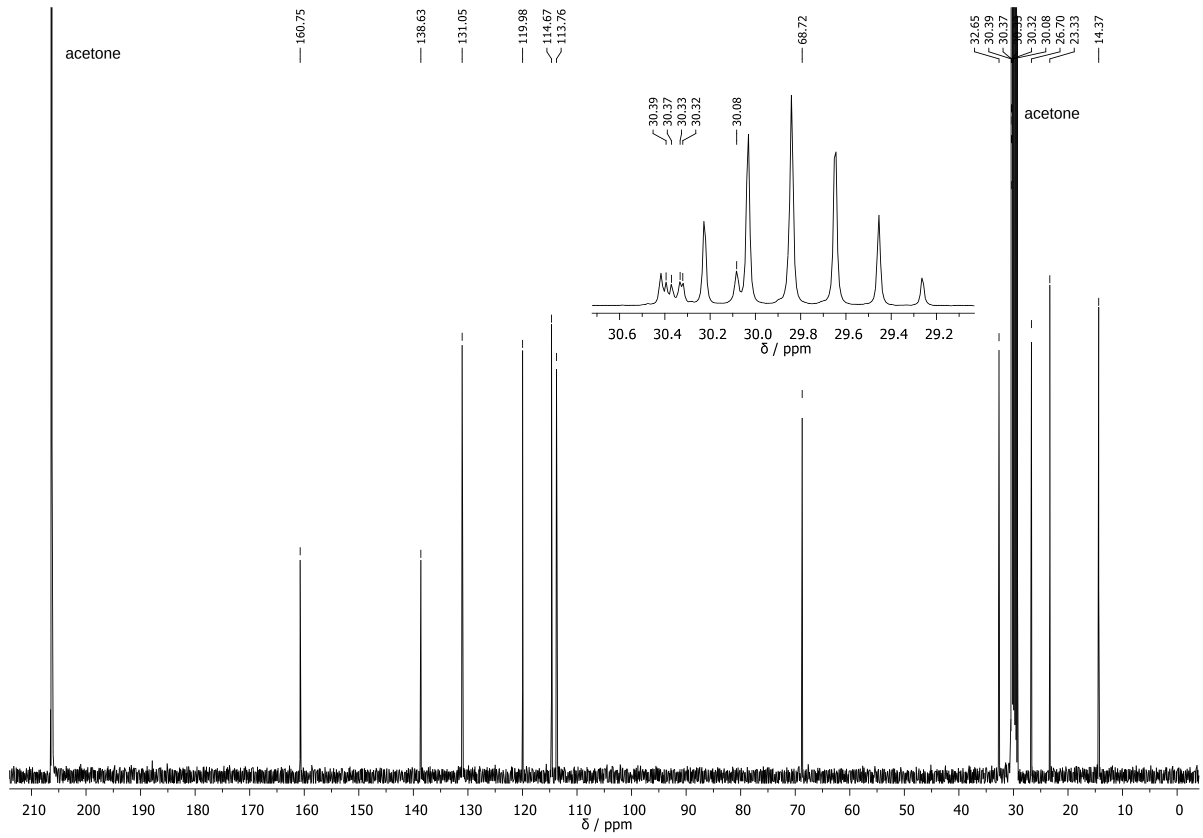

Figure S4: ^{13}C- NMR spectrum (101 MHz, 298 K, acetone-d_6) of the alkylated disulphide (**4**).

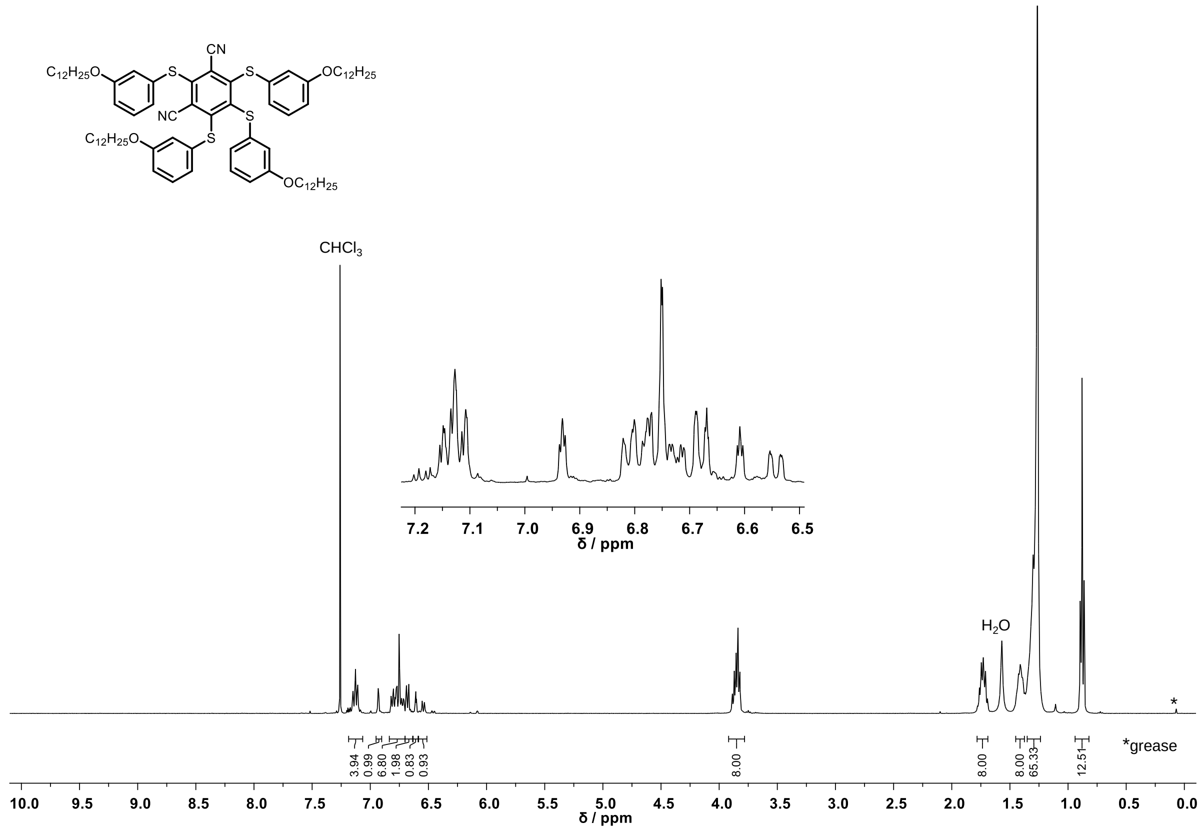

Figure S5: ^1H- NMR spectrum (400 MHz, 298 K, $CDCl_3$) of ***mm*OC$_{12}$**.

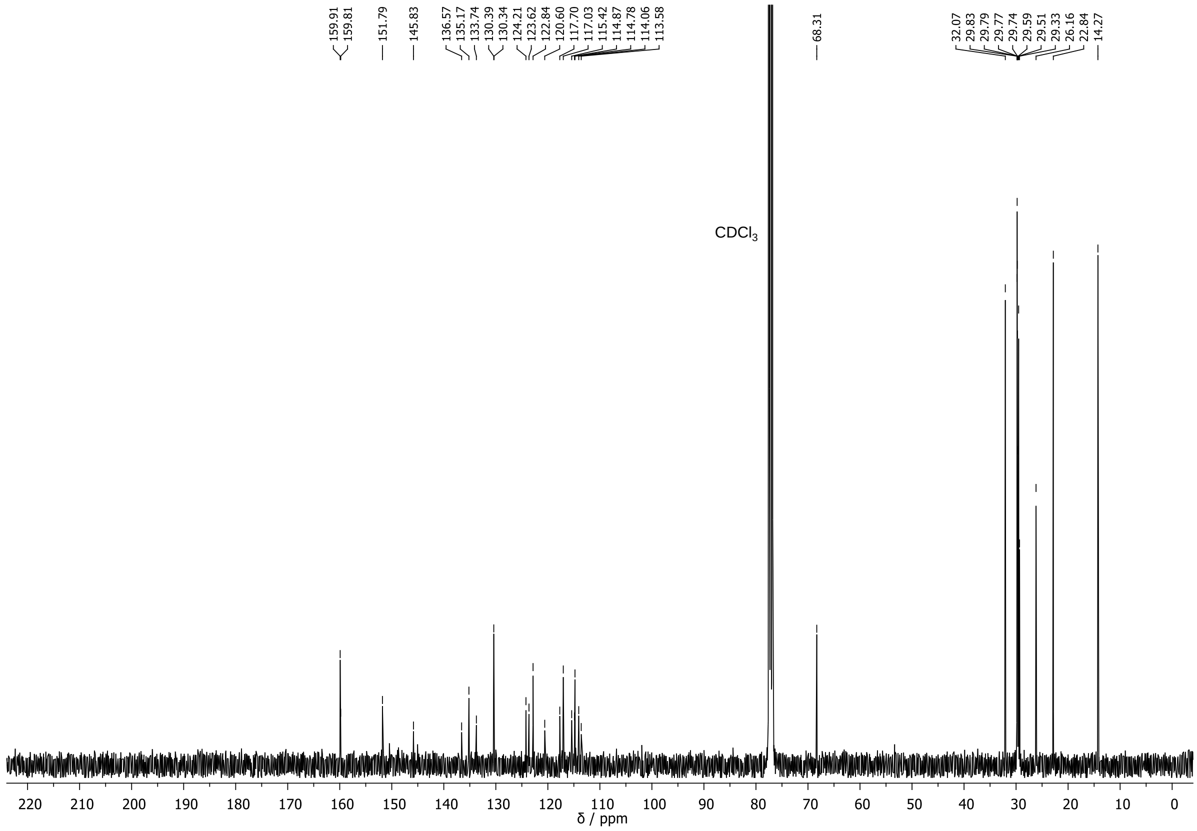

Figure S6: ^{13}C- NMR spectrum (101 MHz, 298 K, $CDCl_3$) of ***mm*OC$_{12}$**.

General Procedures

MSNs with Luminophore

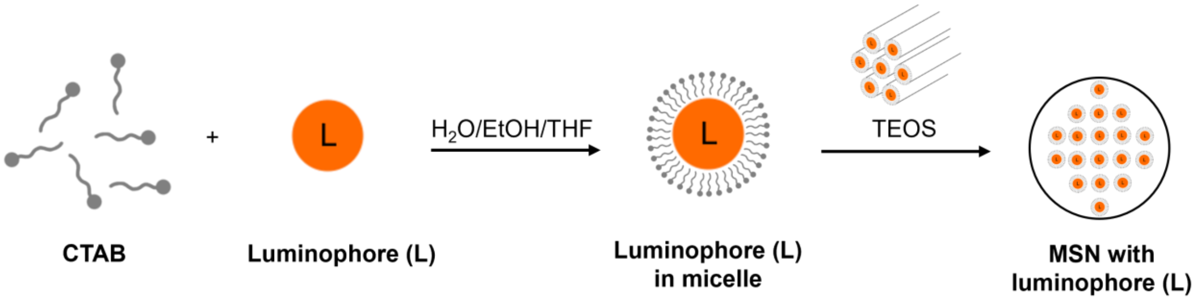

Scheme S2: General scheme for the synthesis of luminescent MSNs (modified after Lasic and Donato *et al.*[2,3]). First, the luminophore is incorporated into micelles, which then probably form hexagonal aligned (2D or 3D) micelles, which serve as a template for the formation of the MSNs.[4]

MSNs with luminophore were synthesised via a *Stöber*-like synthesis.[3,5,6] The luminophore (5 mg) was dissolved in 2 mL distilled THF (***pp*OC$_{12}$**) or ethanol (***mm*OC$_{12}$**) and added to a solution of cetyltrimethylammonium bromide (CTAB, 80 mg, 0.220 mmol) and tris(hydroxymethyl)aminomethane (TRISMA, 100 mg, 0.825 mmol) in 15 mL ultrapure water. After 15 minutes of stirring, tetraethyl orthosilicate (TEOS, 700 µL, 658 mg, 3.269 mmol) was added rapidly to the mixture. The reaction mixture was stirred heavily (750 rpm) overnight at room temperature. After centrifugation, washing several times with water/ethanol and drying in vacuo, a fine powder was received.

MSNs without Luminophore (*Stöber* process)

MSN without luminophore were synthesised via *Stöber* synthesis.[5,6] At first a solution of 80 mg CTAB and 100 mg TRISMA in 15 mL of water was prepared. Under rapid stirring (750 rpm) TEOS (700 µL, 658 mg, 3.269 mmol) was added to the solution. After stirring overnight, centrifugation, washing and drying in vacuum a fine white powder was received.

Thin Films

Thin films were made via drop casting on a glass sheet. 2 w.% MSNs were dispersed in a saturated solution of PVA in water. Then, the polymer mixture was given dropwise on a glass slide, until the slide was completely covered. After that, the slide was heated on a heating plate at 60 °C until the solvent was completely evaporated.

Electrospinning

PVA (polyvinyl alcohol, 87-89 % hydrolysed, 146-186 kDa) membranes were created from solutions in DIW (deionised water). For parameter optimisation PVA (0.71 g, 3.4 wt.%) was dissolved in DIW (20 mL) overnight at 85 °C. Solutions including MSNs (10 wt.%) were prepared by dissolving PVA (0.94 g, 4.5 wt.%) in DIW (20 mL). MSNs (58.7 mg) were suspended in DIW (2 mL) using an ultrasonic bath for 5 min. Under vigorous stirring the PVA solution (4.5 wt.%, 11 mL) was slowly added, further diluting the PVA to 3.8 wt.%.

Inscribing in NFs

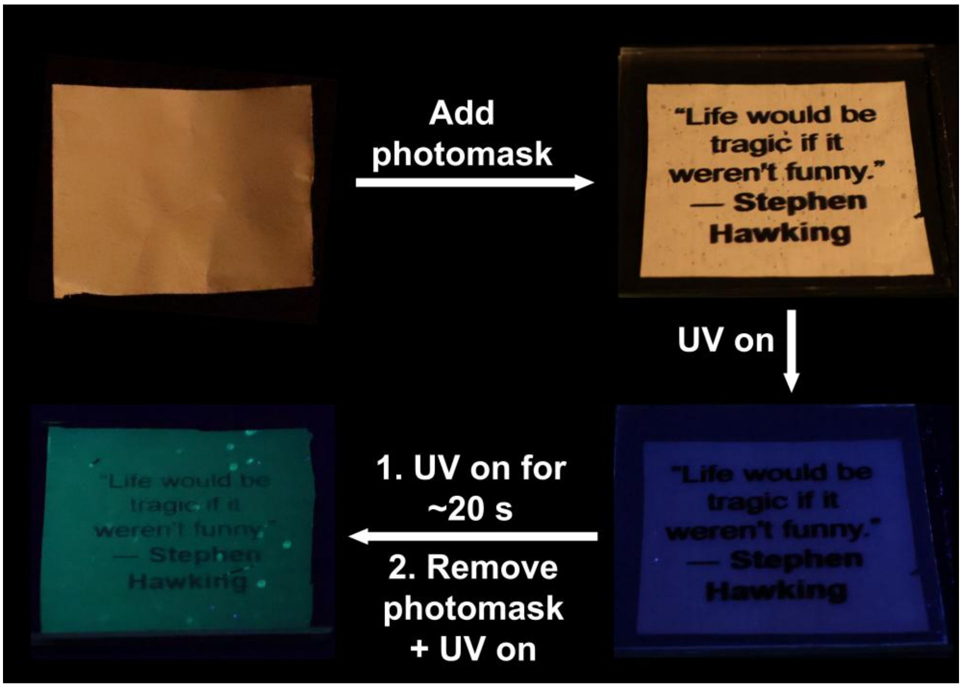

Scheme S3: Schematic depiction of the inscription process into the NFs.

A square piece of the electrospun fleece with ***mm*OC$_{12}$** MSNs is placed between two glass slides (5 x 5 cm) and a photomask, printed on a transparent foil, is placed on top of the fleece. Then, the fleece is irradiated with a UV pocket lamp (395 nm) for approximately 20 s. After that, the lamp is switched off and the inscription is finished. The imprinted image/script can be read out by applying UV light again.

SEM Imaging

MSNs

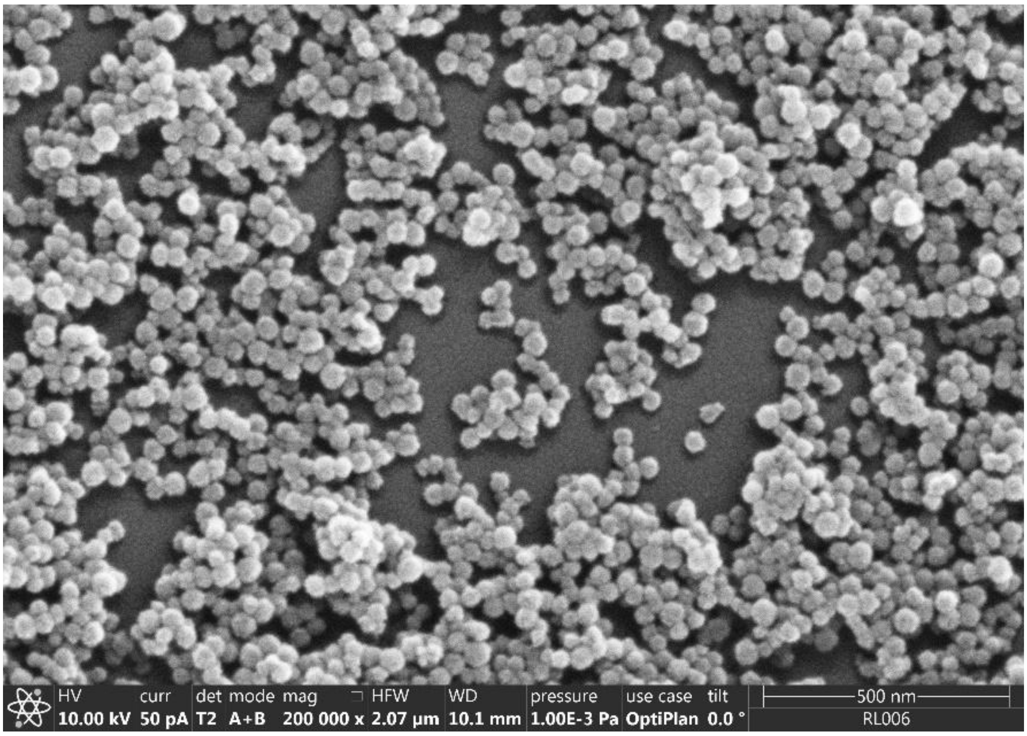

Figure S7: SEM image of MSNs without luminophore.

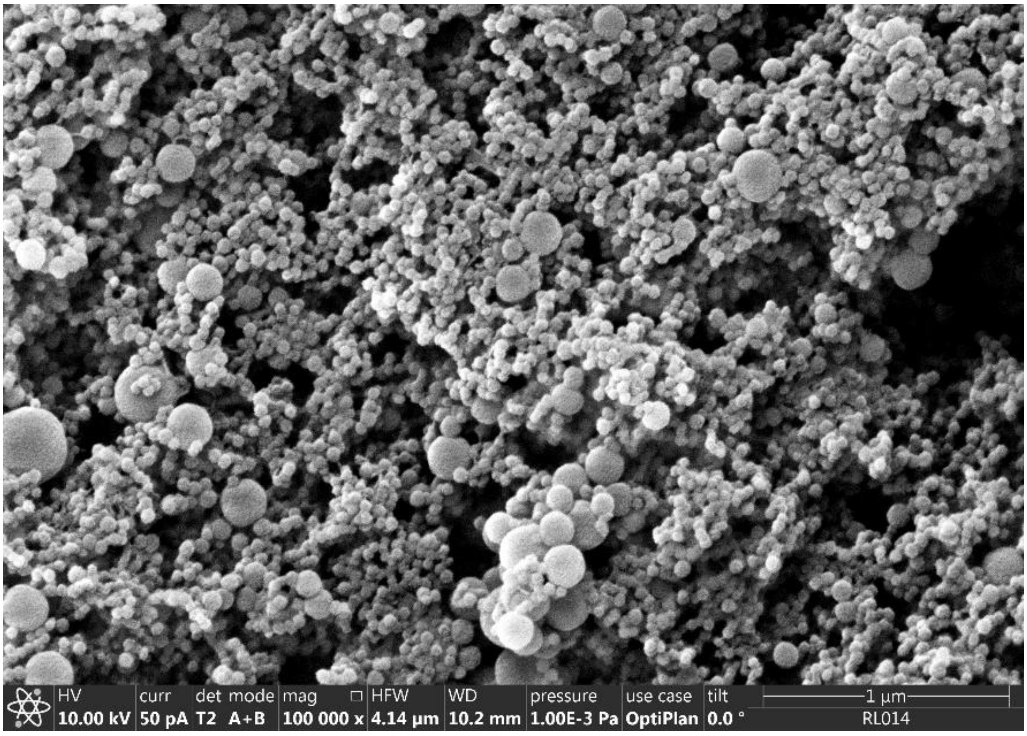

Figure S8: SEM image of MSNs with ***pp*OC₁₂** luminophore.

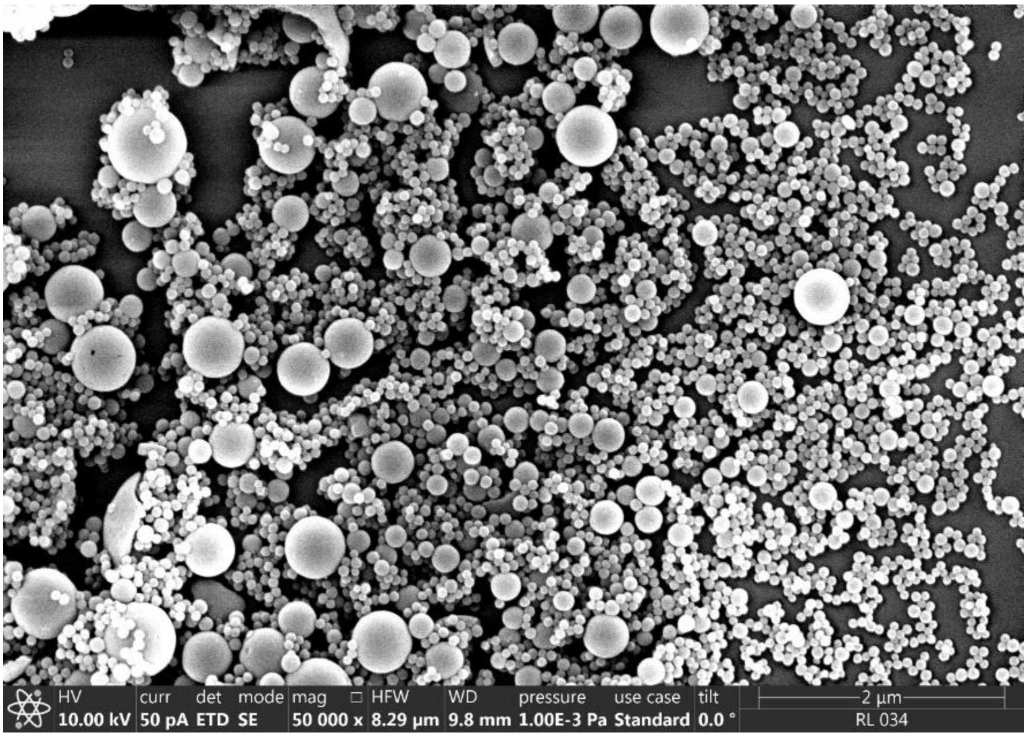

Figure S9: SEM image of MSNs with ***mm*OC$_{12}$** luminophore.

MSNs in 3D Material

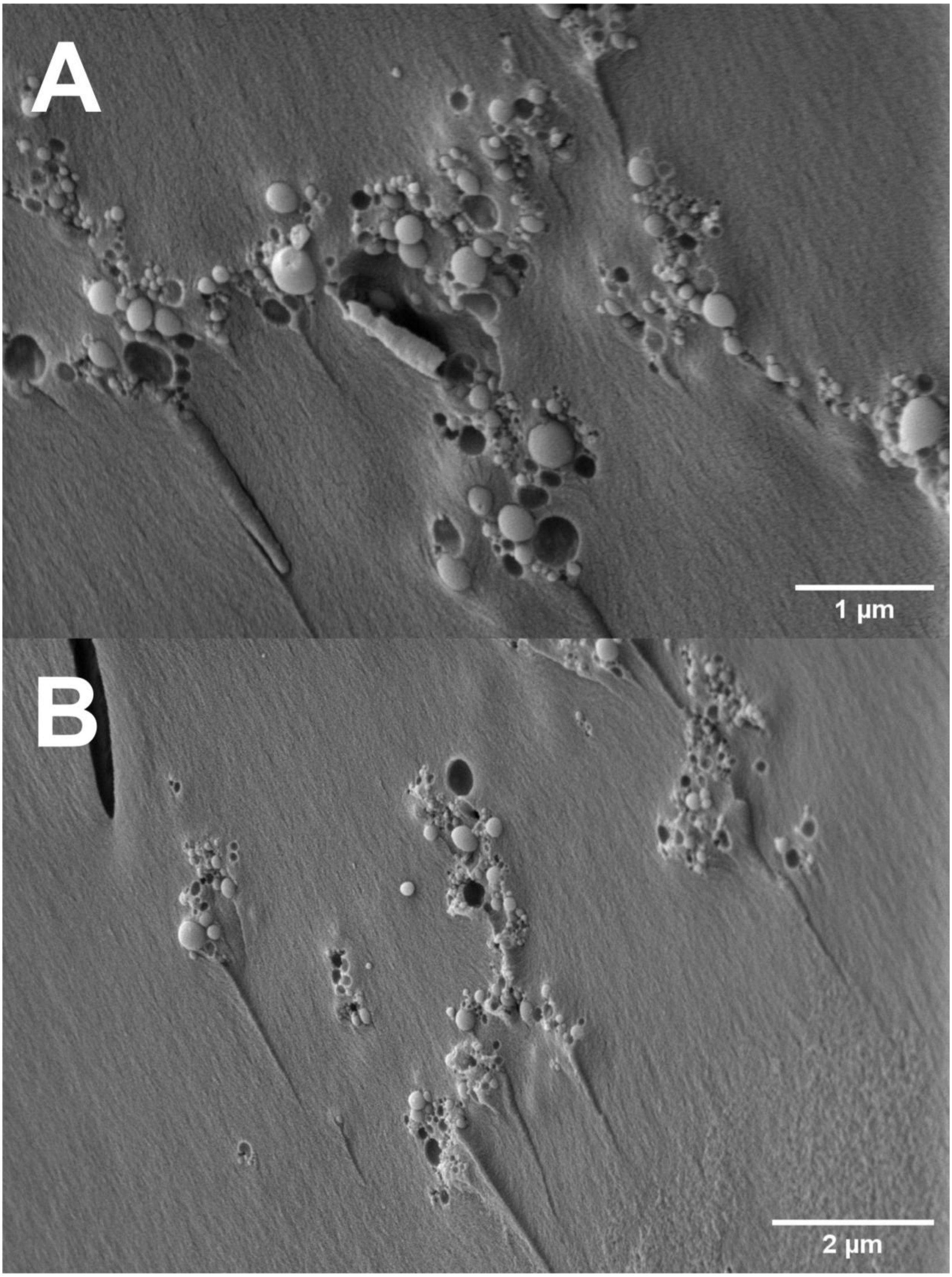

Figure S10: A/B) SEM images of MSNs in 3D material on the breaking edge of the object.

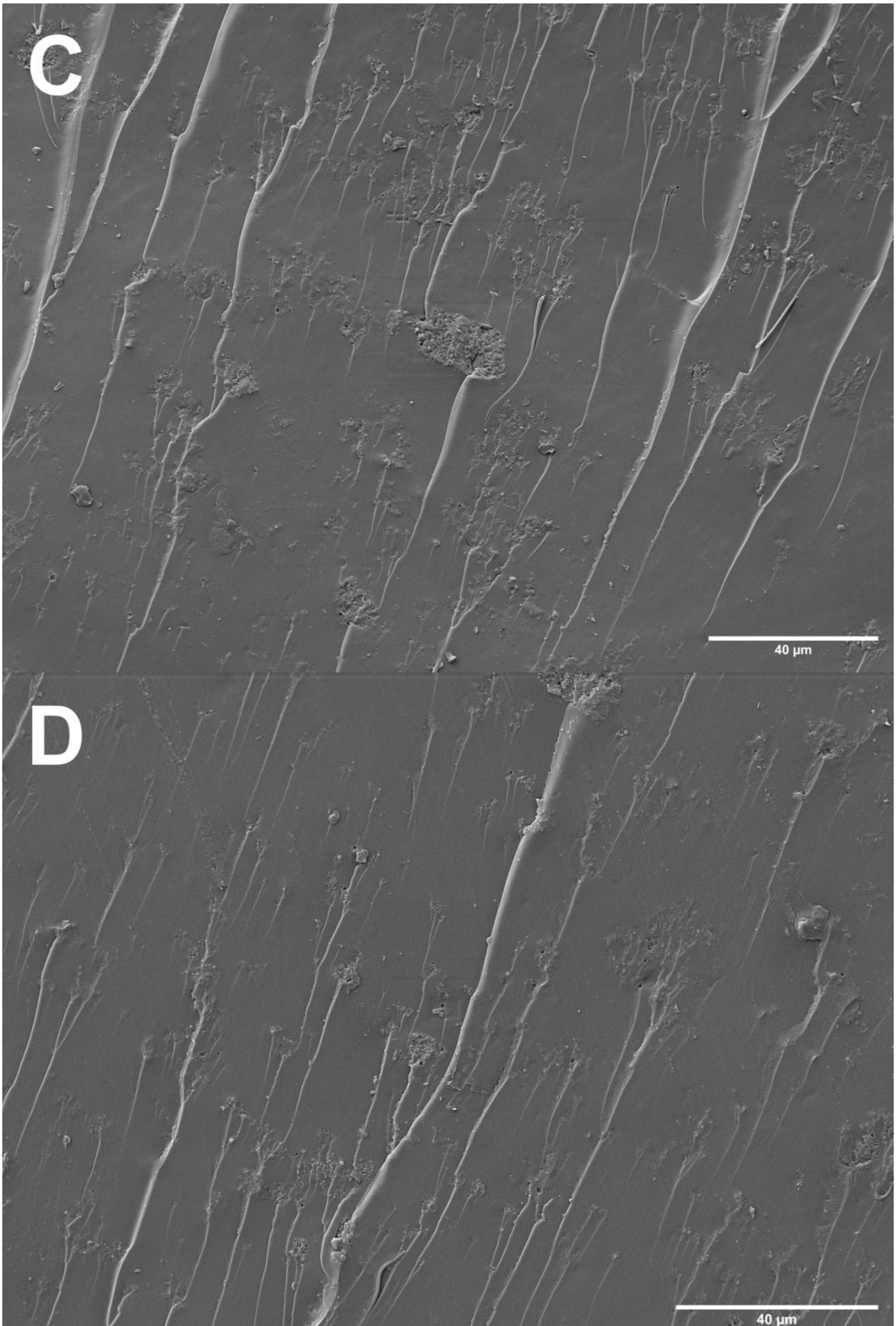

Figure S11: C/D) SEM images of MSNs in 3D material on a breaking edge of the object.

MSNs in PVA Nanofibers

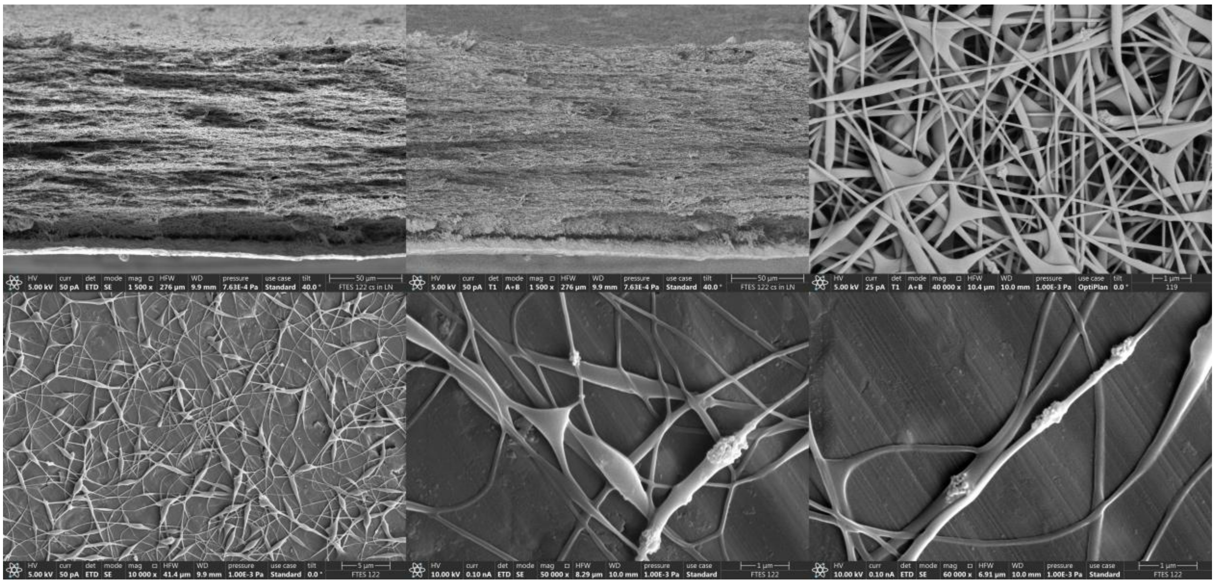

Figure S12: SEM images of the PVA nanofibers with ***mm*OC$_{12}$** MSNs with different magnifications. The upper images were taken from a thick fleece, while the images below were taken from a thin layer of fibers.

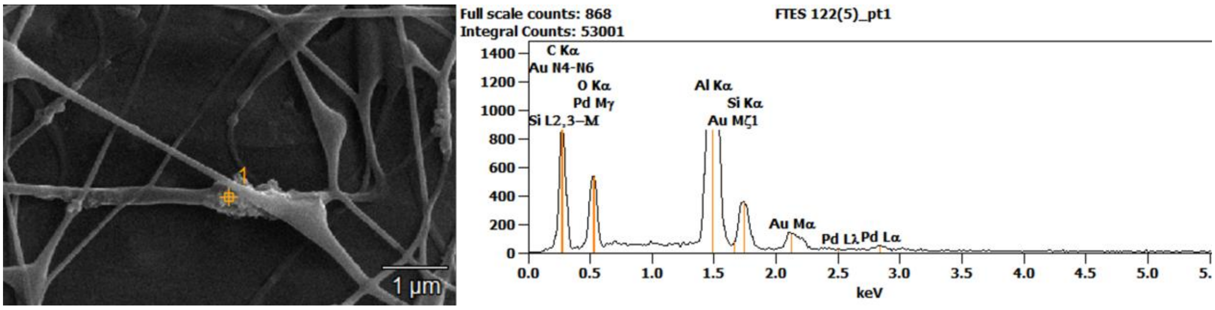

Figure S13: SEM image and EDX spectrum from a NF with ***mm*OC$_{12}$** MSNs.

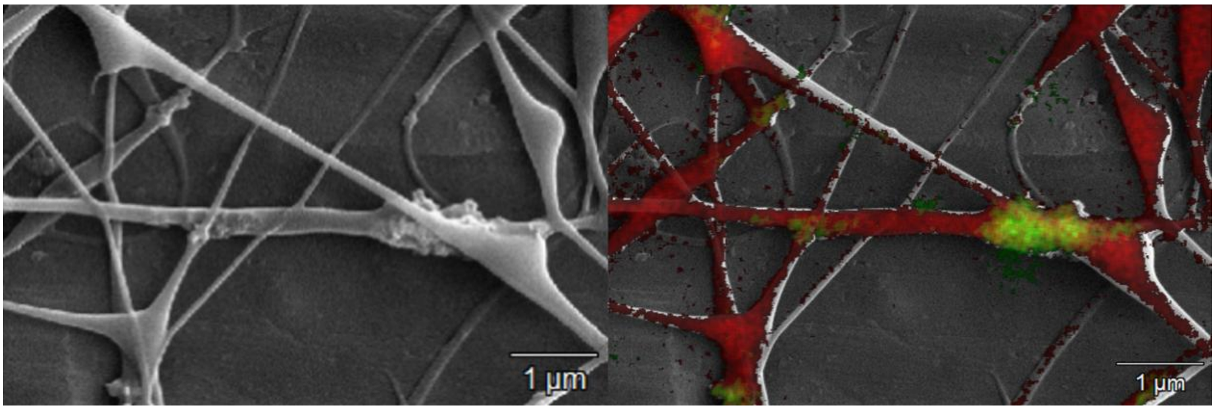

Figure S14: SEM image of the NFs with ***mm*OC$_{12}$** MSNs (left) and the same image overlayed with a spatially resolved EDX surface measurement, red corresponding to the C Kα signal and green representing the Si Kα response (right).

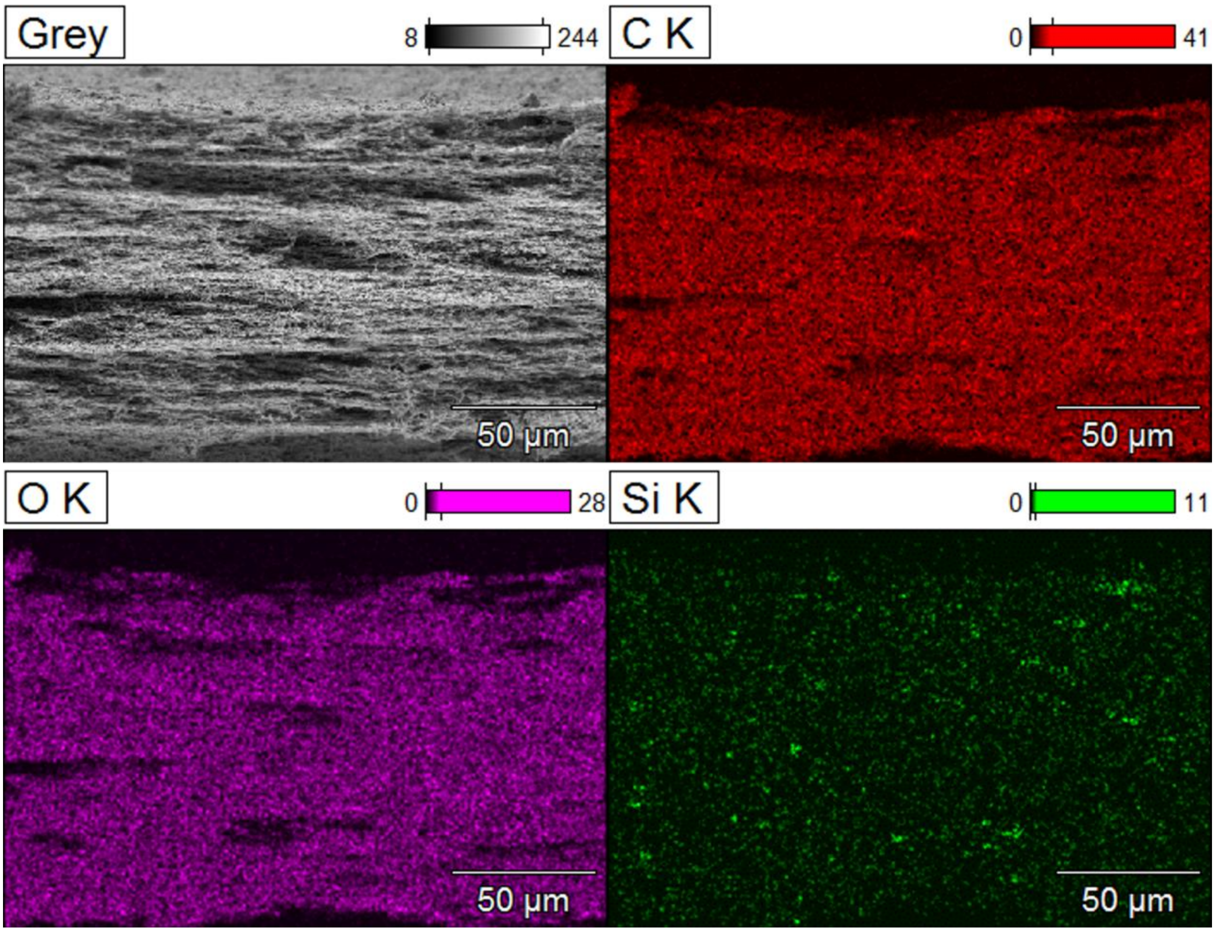

Figure S15: SEM image of the PVA fleece with ***mm*OC$_{12}$** MSNs (upper left), spatially resolved EDX surface measurement from the C Kα signal (upper right), spatially resolved EDX surface measurement from the O Kα signal (bottom left) and spatially resolved EDX surface measurement from the Si Kα signal (bottom right). The Si Kα measurement depicts an even distribution of the MSNs in the NF fleece, while the C and O signals can represent the polymer in the NFs.

Afterglow Experiments

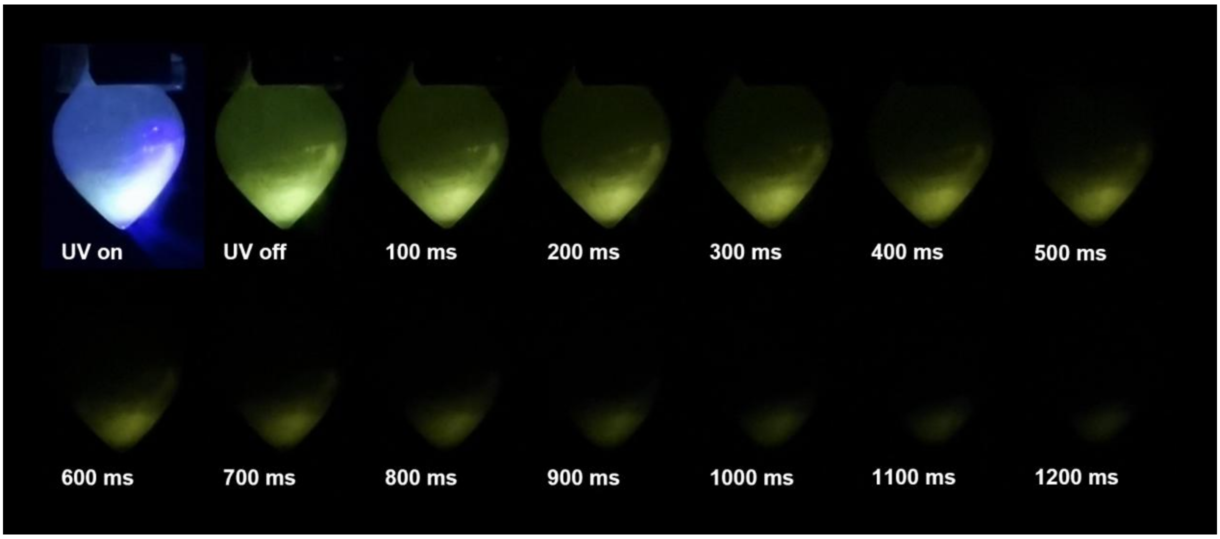

Figure S16: Afterglow of irradiated MSNs with ***mm*OC$_{12}$** in aqueous suspension at 77 K upon irradiation with a 365 nm LED pocket lamp.

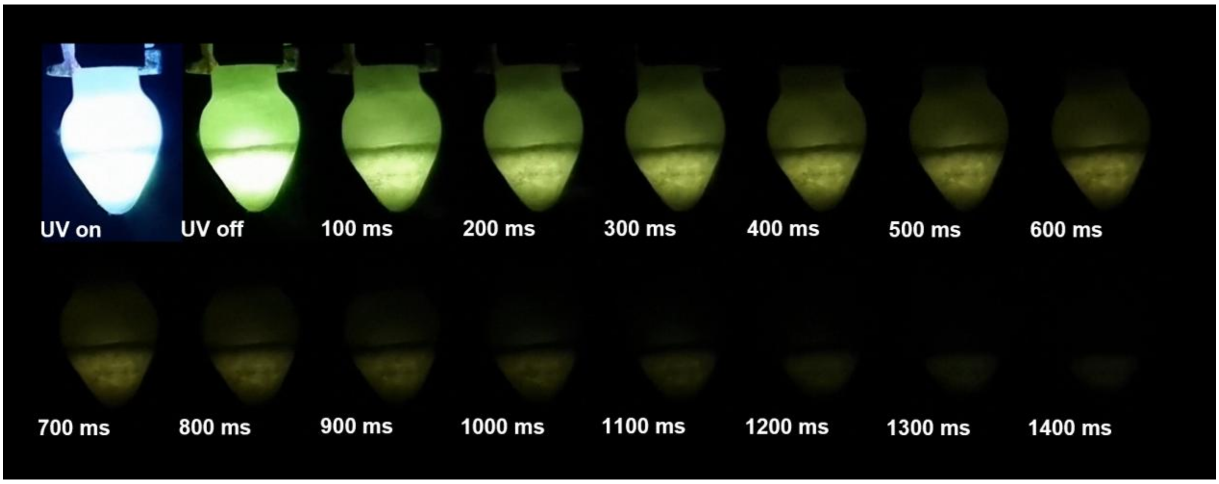

Figure S17: Afterglow of irradiated MSNs with ***mm*OC$_{12}$** in methanolic suspension at 77 K upon irradiation with a 365 nm LED pocket lamp.

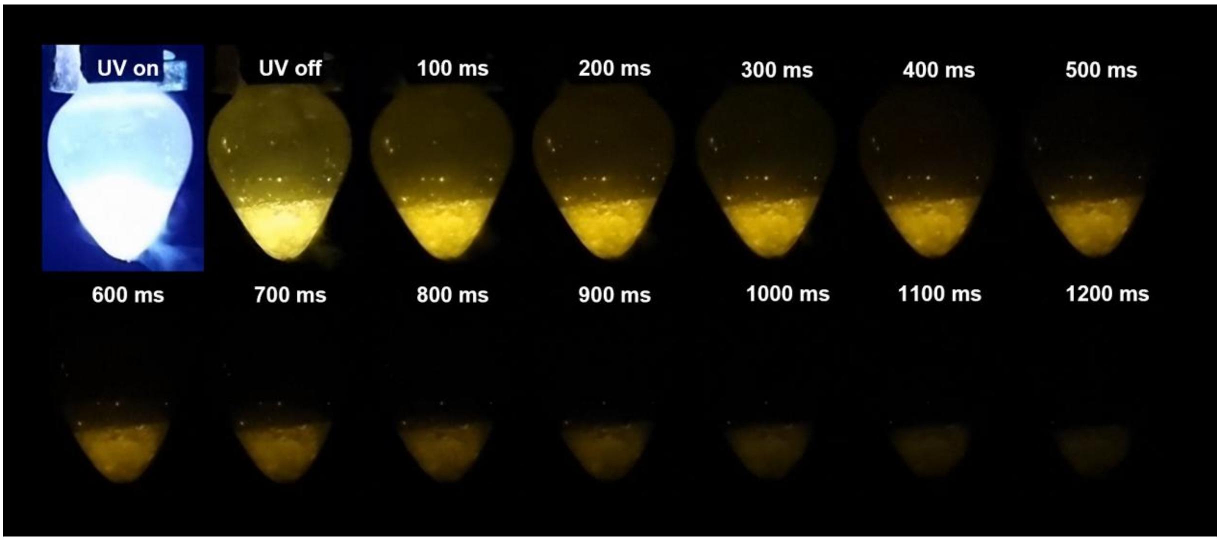

Figure S18: Afterglow of irradiated MSNs with ***mm*OC$_{12}$** in glycerolic suspension at 77 K upon irradiation with a 365 nm LED pocket lamp.

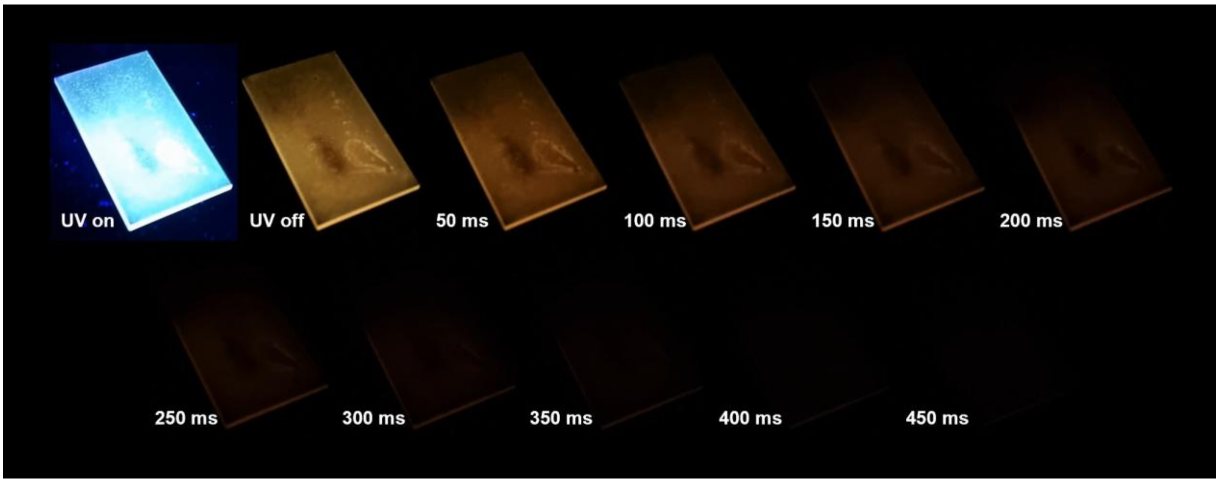

Figure S19: Afterglow of irradiated MSNs with ***mm*OC$_{12}$** in PVA (87-89 % hydrolysed) film at room temperature upon irradiation with a 365 nm LED pocket lamp.

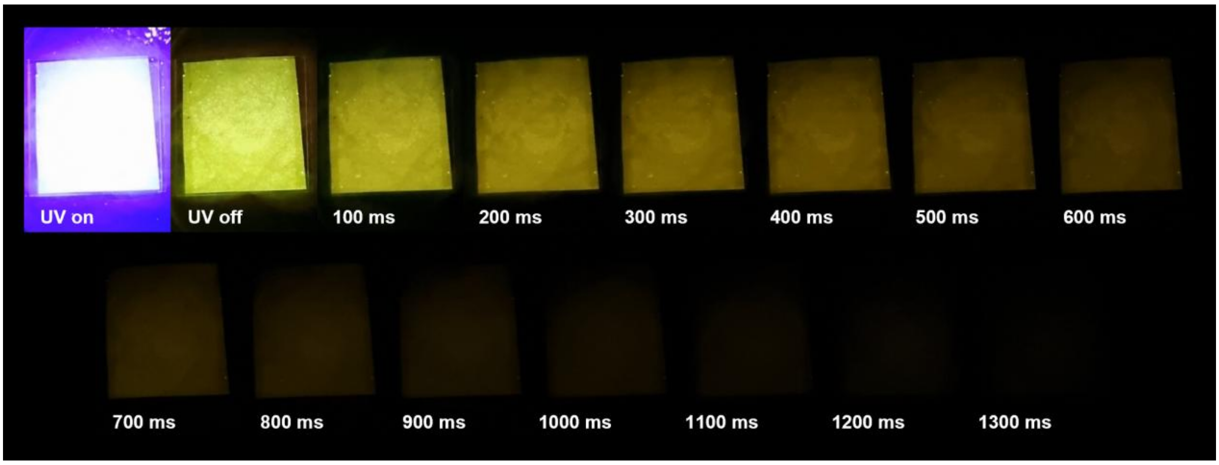

Figure S20: Afterglow of irradiated MSNs with ***mm*OC$_{12}$** in electrospun PVA (87-89 % hydrolysed) NFs at 77 K upon irradiation with a 365 nm LED pocket lamp.

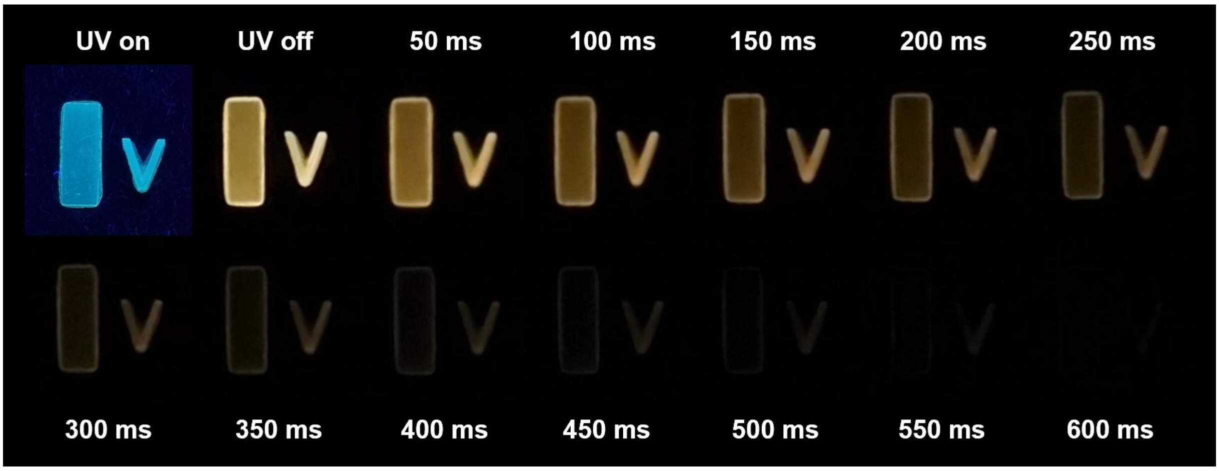

Figure S21: Afterglow of irradiated MSNs with ***mm*OC$_{12}$** 3D printed objects at room temperature upon irradiation with a 365 nm LED pocket lamp.

Photographs of MSN Powders and 3D objects

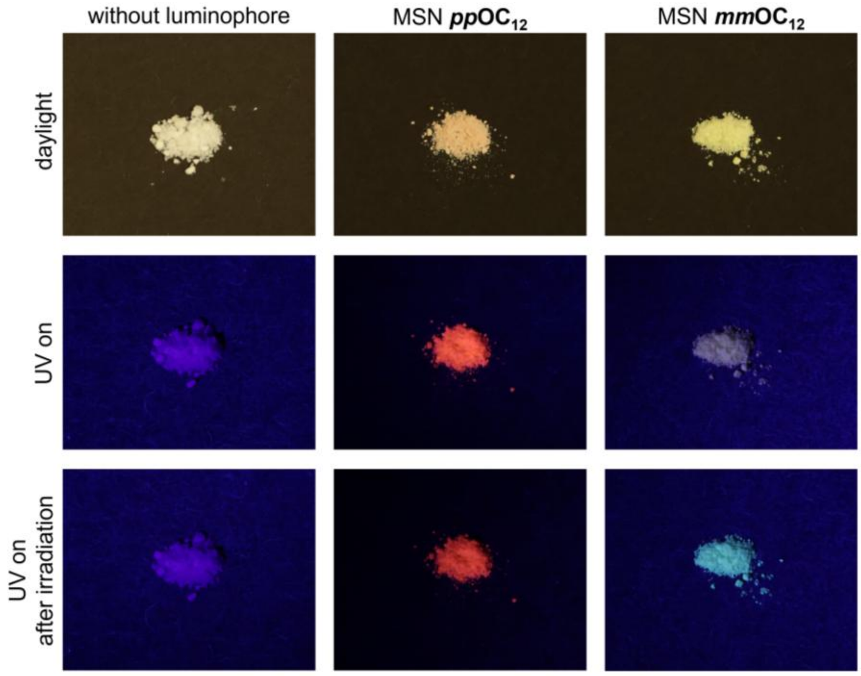

Figure S22: MSN powder under daylight and UV light ((λ_{exc} = 365 nm), as well after irradiation with UV (λ_{exc} = 365 nm).

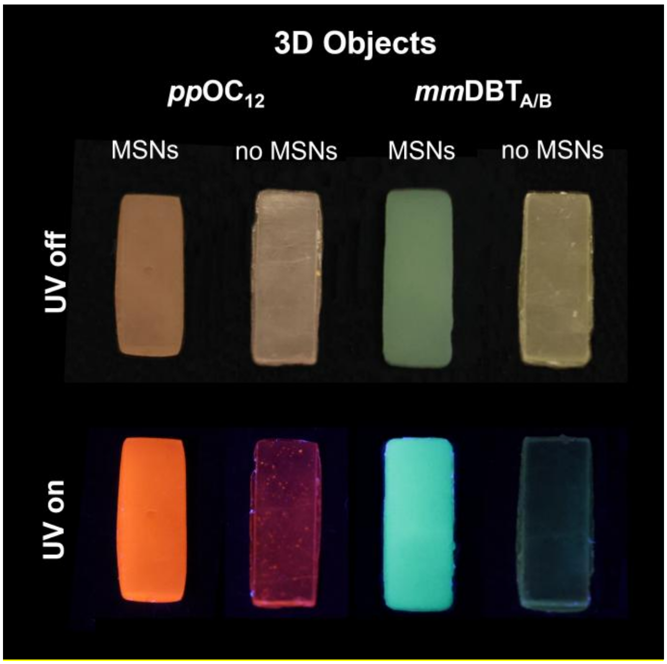

Figure S23: Comparison between 3D objects doped with solely luminophore and the luminophore within MSNs for ***pp*OC$_{12}$** (left) and ***mm*OC$_{12}$** (right). The samples are shown under daylight conditions (top) and upon irradiation with a 365 nm LED pocket lamp (bottom). To ensure comparability, the amount of the sole luminophore added was adjusted to the amount of luminophore incorporated in the 2 wt.% MSNs. The printed strips have a dimension of 13 x 5 mm.

Photophysical Characterisation

Lifetimes and Quantum Yields

Table S1: Summary of photophysical data for the MSNs in 3D material and as powder.

Sample	λ_{em} [nm]	τ_{av_amp}	k_r [c] [s^{-1}]	k_{nr} [c] [s^{-1}]	Φ_L [b] ± 2 [%]
**3D material with *mm*DBT$_{A/B}$ MSNs**	494	36.3 ± 0.4 ms [d]	1.6 ± 0.6	25.9 ± 0.7	6
***mm*DBT$_{A/B}$ MSN powder**	503	4.85 ± 0.06 ns [d]	(8 ± 4) x 10^6	(197 ± 5) x 10^6	4
**3D material with *pp*OC$_{12}$ MSNs**	592	6.85 ± 0.02 µs [e]	< 5.83 x 10^3	(1.40 < k_{nr}< 1.45) x 10^5	<2
***pp*OC$_{12}$ MSN powder**	600	5.34 ± 0.04 µs [e]	(0.7 ± 0.4) x 10^4	(17.9 ± 0.4) x 10^4	4

[a] The lifetimes were obtained by using a laser diode λ_{exc} = 376 nm. For multi-exponential decays, the amplitude-weighted average lifetimes (τ_{av_amp}) are shown.[7]

[b] Quantum yields were measured with λ_{exc} = 350 nm.

[c] The rate average constants were determined according to $k_r = \Phi_L / \tau$ and $k_{nr} = (1- \Phi_L) / \tau$. [7] Detailed information can be found in the Figures S24-S29.

[d] Tri-exponential decays.

[e] Bi-exponential decays.

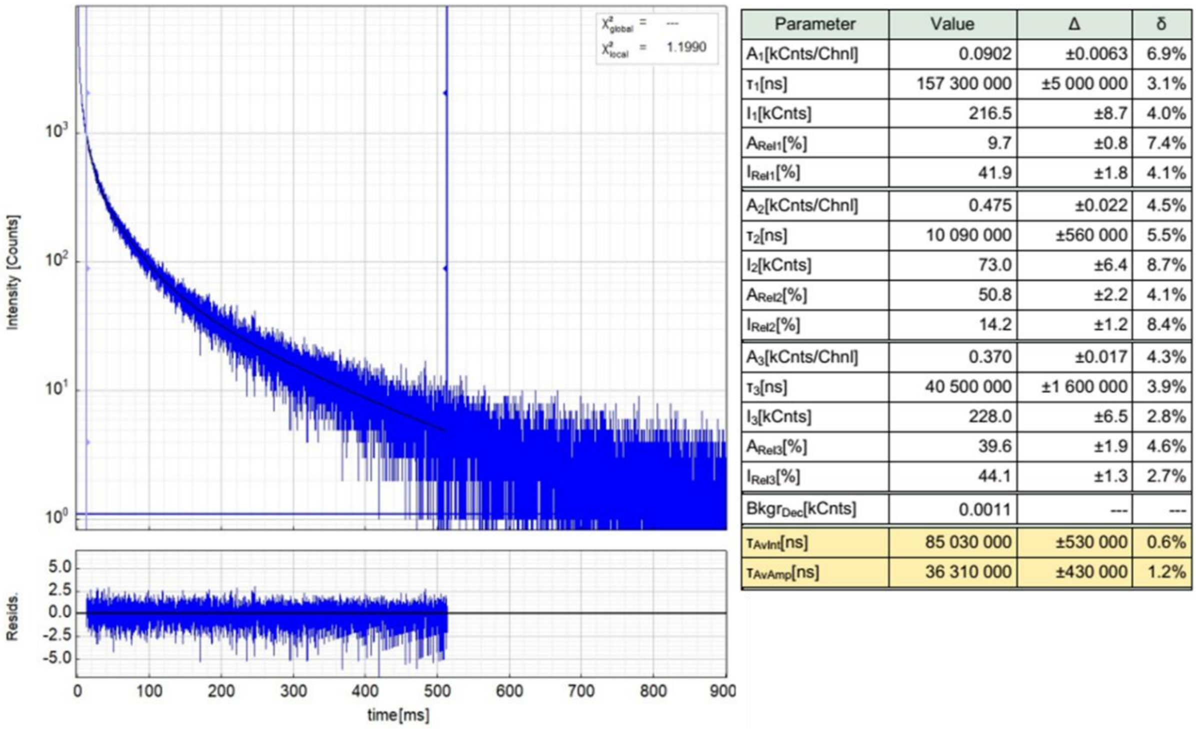

Parameter	Value	Δ	δ
A_1[kCnts/Chnl]	0.0902	±0.0063	6.9%
τ_1[ns]	157 300 000	±5 000 000	3.1%
I_1[kCnts]	216.5	±8.7	4.0%
A_{Rel1}[%]	9.7	±0.8	7.4%
I_{Rel1}[%]	41.9	±1.8	4.1%
A_2[kCnts/Chnl]	0.475	±0.022	4.5%
τ_2[ns]	10 090 000	±560 000	5.5%
I_2[kCnts]	73.0	±6.4	8.7%
A_{Rel2}[%]	50.8	±2.2	4.1%
I_{Rel2}[%]	14.2	±1.2	8.4%
A_3[kCnts/Chnl]	0.370	±0.017	4.3%
τ_3[ns]	40 500 000	±1 600 000	3.9%
I_3[kCnts]	228.0	±6.5	2.8%
A_{Rel3}[%]	39.6	±1.9	4.6%
I_{Rel3}[%]	44.1	±1.3	2.7%
$Bkgr_{Dec}$[kCnts]	0.0011	---	---
τ_{AvInt}[ns]	85 030 000	±530 000	0.6%
τ_{AvAmp}[ns]	36 310 000	±430 000	1.2%

Figure S24: Left: Raw (experimental) time-resolved photoluminescence decay of the 3D material with ***mm*****DBT$_{A/B}$** MSNs at 298 K, including the residuals (λ_{exc} = 376 nm, λ_{em} = 494 nm). Right: Fitting parameters including pre-exponential factors and confidence limits.

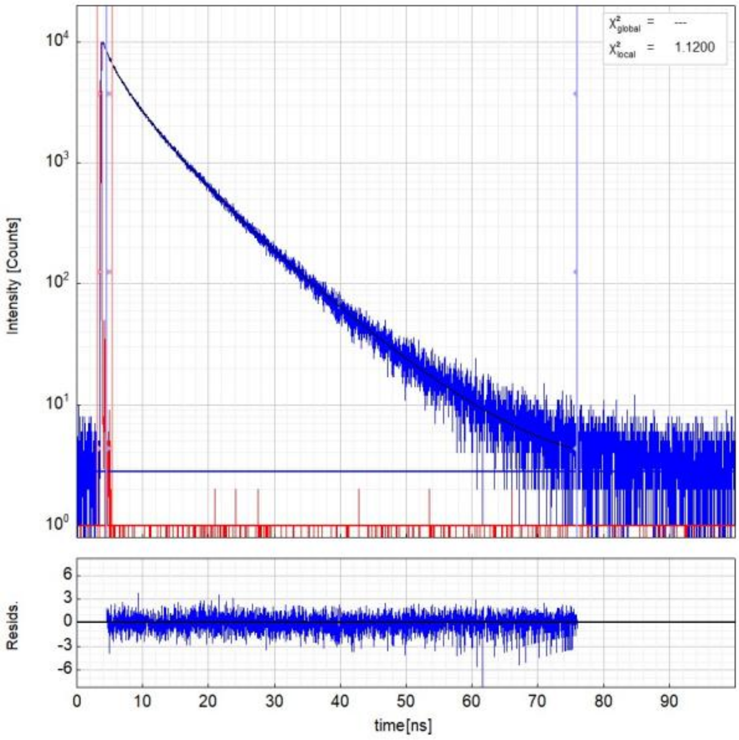

Parameter	Value	Δ	δ
A_1[kCnts/Chnl]	2.48	±0.35	14%
τ_1[ns]	9.72	±0.31	3.1%
I_1[kCnts]	1 510	±180	11%
A_{Rel1}[%]	22.7	±3.0	13%
I_{Rel1}[%]	45.4	±5.2	11%
A_2[kCnts/Chnl]	4.91	±0.12	2.4%
τ_2[ns]	4.86	±0.39	7.9%
I_2[kCnts]	1 500	±110	7.2%
A_{Rel2}[%]	45.0	±1.0	2.2%
I_{Rel2}[%]	45.0	±3.3	7.3%
A_3[kCnts/Chnl]	3.55	±0.25	6.9%
τ_3[ns]	1.45	±0.17	11%
I_3[kCnts]	321	±61	19%
A_{Rel3}[%]	32.5	±2.7	8.2%
I_{Rel3}[%]	9.7	±1.9	19%
$Bkgr_{Dec}$[kCnts]	0.0028	---	---
$Bkgr_{IRF}$[Cnts/Chnl]	1.0000	---	---
$Shift_{IRF}$[ps]	-4.0000	---	---
τ_{AvInt}[ns]	6.733	±0.015	0.2%
τ_{AvAmp}[ns]	4.854	±0.057	1.2%

Figure S25: Left: Raw (experimental) time-resolved photoluminescence decay of the ***mm*****DBT$_{A/B}$** MSN powder and the instrument response function (red) at 298 K, including the residuals (λ_{exc} = 376 nm, λ_{em} = 503 nm). Right: Fitting parameters including pre-exponential factors and confidence limits.

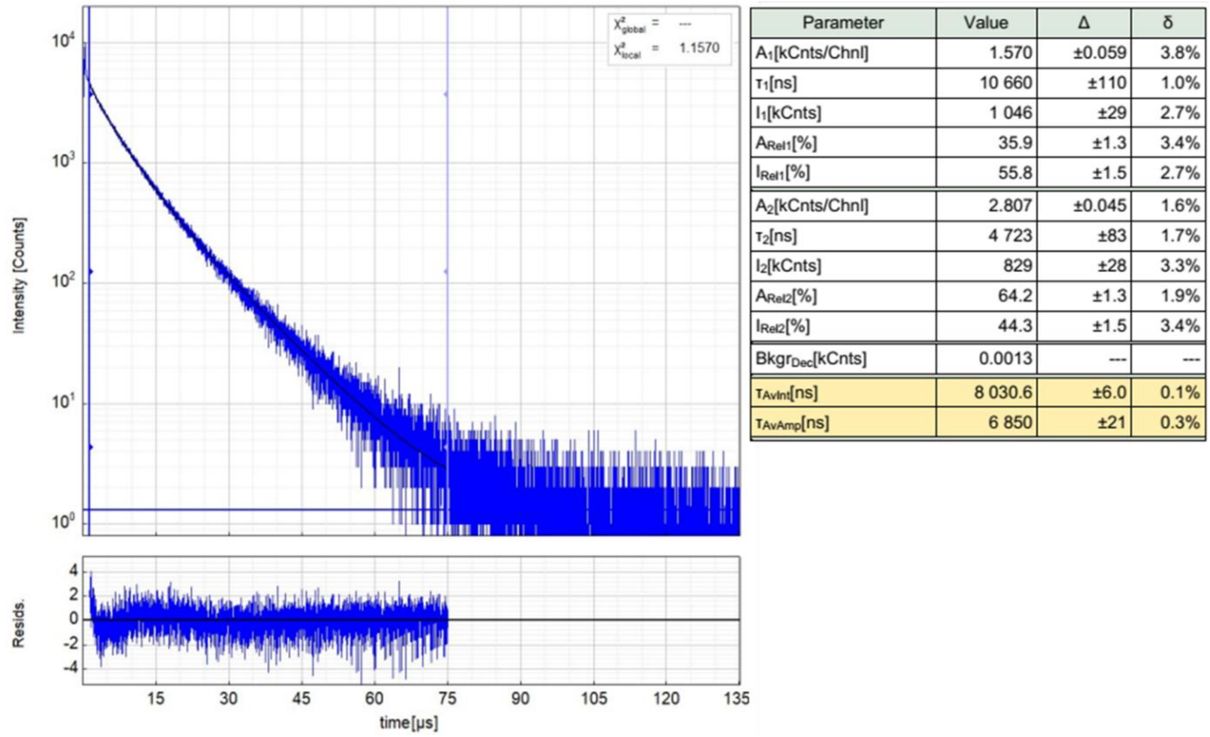

Parameter	Value	Δ	δ
A_1[kCnts/Chnl]	1.570	±0.059	3.8%
τ_1[ns]	10 660	±110	1.0%
I_1[kCnts]	1 046	±29	2.7%
A_{Rel1}[%]	35.9	±1.3	3.4%
I_{Rel1}[%]	55.8	±1.5	2.7%
A_2[kCnts/Chnl]	2.807	±0.045	1.6%
τ_2[ns]	4 723	±83	1.7%
I_2[kCnts]	829	±28	3.3%
A_{Rel2}[%]	64.2	±1.3	1.9%
I_{Rel2}[%]	44.3	±1.5	3.4%
$Bkgr_{Dec}$[kCnts]	0.0013	---	---
τ_{AvInt}[ns]	8 030.6	±6.0	0.1%
τ_{AvAmp}[ns]	6 850	±21	0.3%

Figure S26: Left: Raw (experimental) time-resolved photoluminescence decay of the 3D material with ***pp*OC$_{12}$** MSNs at 298 K, including the residuals (λ_{exc} = 376 nm, λ_{em} = 592 nm). Right: Fitting parameters including pre-exponential factors and confidence limits.

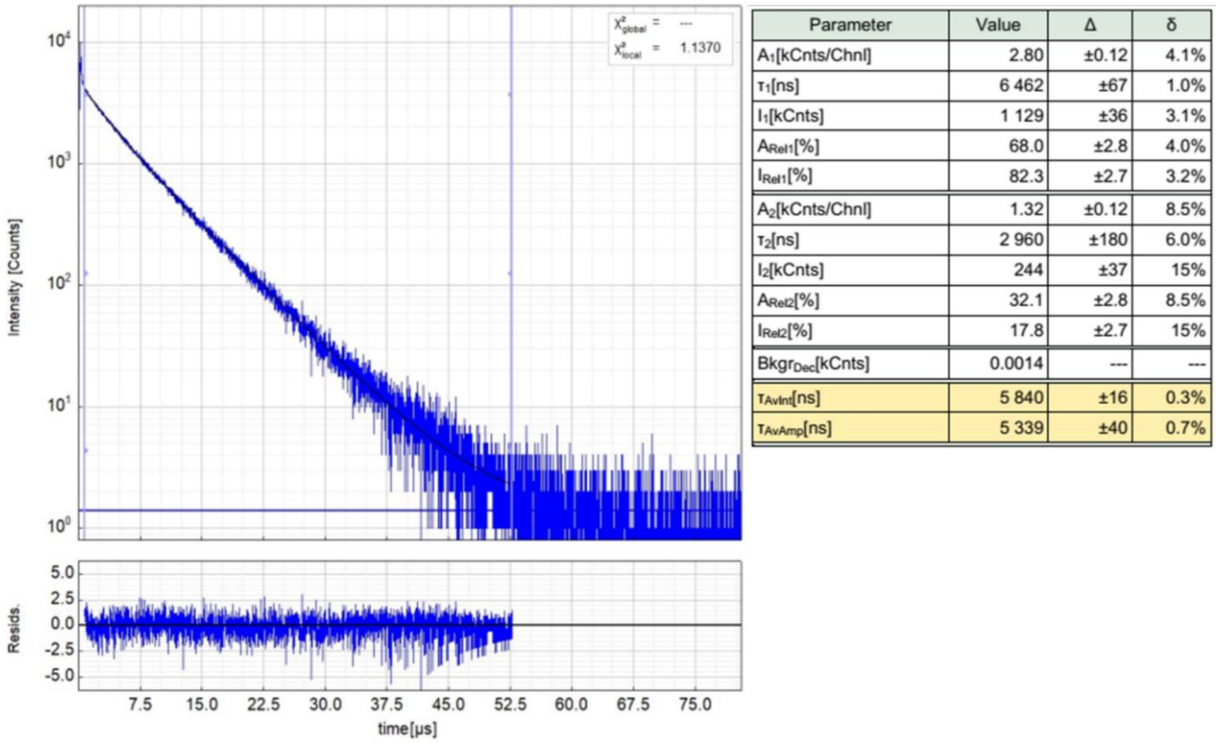

Parameter	Value	Δ	δ
A_1[kCnts/Chnl]	2.80	±0.12	4.1%
τ_1[ns]	6 462	±67	1.0%
I_1[kCnts]	1 129	±36	3.1%
A_{Rel1}[%]	68.0	±2.8	4.0%
I_{Rel1}[%]	82.3	±2.7	3.2%
A_2[kCnts/Chnl]	1.32	±0.12	8.5%
τ_2[ns]	2 960	±180	6.0%
I_2[kCnts]	244	±37	15%
A_{Rel2}[%]	32.1	±2.8	8.5%
I_{Rel2}[%]	17.8	±2.7	15%
$Bkgr_{Dec}$[kCnts]	0.0014	---	---
τ_{AvInt}[ns]	5 840	±16	0.3%
τ_{AvAmp}[ns]	5 339	±40	0.7%

Figure S27: Left: Raw (experimental) time-resolved photoluminescence decay of the ***pp*OC$_{12}$** MSN powder at 298 K, including the residuals (λ_{exc} = 376 nm, λ_{em} = 600 nm). Right: Fitting parameters including pre-exponential factors and confidence limits.

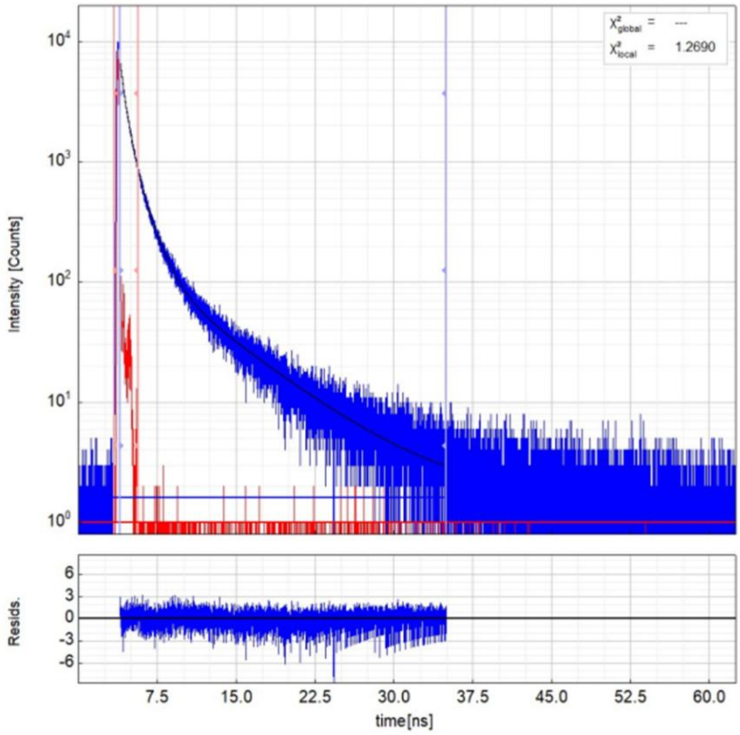

Parameter	Value	Δ	δ
A_1[kCnts/Chnl]	0.1711	±0.0051	3.0%
τ_1[ns]	6.441	±0.079	1.2%
I_1[kCnts]	275.5	±5.1	1.8%
A_{Rel1}[%]	1.7	±0.1	2.1%
I_{Rel1}[%]	13.9	±0.3	1.7%
A_2[kCnts/Chnl]	2.72	±0.12	4.2%
τ_2[ns]	1.291	±0.024	1.8%
I_2[kCnts]	876	±25	2.8%
A_{Rel2}[%]	25.5	±0.9	3.4%
I_{Rel2}[%]	44.1	±1.2	2.7%
A_3[kCnts/Chnl]	7.791	±0.066	0.8%
τ_3[ns]	0.4299	±0.0100	2.3%
I_3[kCnts]	838	±23	2.7%
A_{Rel3}[%]	73.0	±0.9	1.2%
I_{Rel3}[%]	42.2	±1.2	2.8%
$Bkgr_{Dec}$[kCnts]	0.0016	---	---
$Bkgr_{IRF}$[Cnts/Chnl]	1.0000	---	---
$Shift_{IRF}$[ps]	100.0000	---	---
τ_{AvInt}[ns]	1.642	±0.013	0.7%
τ_{AvAmp}[ns]	0.7451	±0.0057	0.8%

Figure S28: Left: Raw (experimental) time-resolved photoluminescence decay of the 3D material with ***pp*****OC$_{12}$** MSNs (photoinitiator signal, see Fig. S34/35) and the instrument response function (red) at 298 K, including the residuals (λ_{exc} = 376 nm, λ_{em} = 460 nm). Right: Fitting parameters including pre-exponential factors and confidence limits.

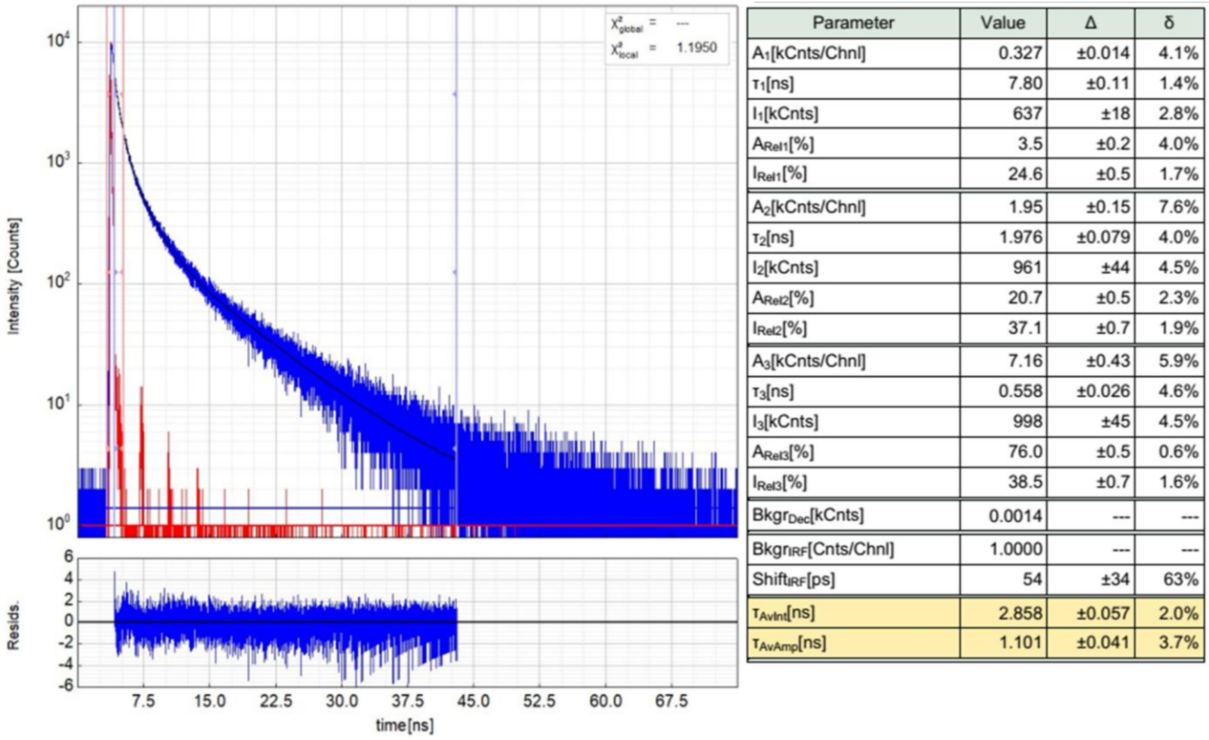

Parameter	Value	Δ	δ
A_1[kCnts/Chnl]	0.327	±0.014	4.1%
τ_1[ns]	7.80	±0.11	1.4%
I_1[kCnts]	637	±18	2.8%
A_{Rel1}[%]	3.5	±0.2	4.0%
I_{Rel1}[%]	24.6	±0.5	1.7%
A_2[kCnts/Chnl]	1.95	±0.15	7.6%
τ_2[ns]	1.976	±0.079	4.0%
I_2[kCnts]	961	±44	4.5%
A_{Rel2}[%]	20.7	±0.5	2.3%
I_{Rel2}[%]	37.1	±0.7	1.9%
A_3[kCnts/Chnl]	7.16	±0.43	5.9%
τ_3[ns]	0.558	±0.026	4.6%
I_3[kCnts]	998	±45	4.5%
A_{Rel3}[%]	76.0	±0.5	0.6%
I_{Rel3}[%]	38.5	±0.7	1.6%
$Bkgr_{Dec}$[kCnts]	0.0014	---	---
$Bkgr_{IRF}$[Cnts/Chnl]	1.0000	---	---
$Shift_{IRF}$[ps]	54	±34	63%
τ_{AvInt}[ns]	2.858	±0.057	2.0%
τ_{AvAmp}[ns]	1.101	±0.041	3.7%

Figure S29: Left: Raw (experimental) time-resolved photoluminescence decay of the 3D material with MSNs without luminophore (photoinitiator signal, see Fig. S34/35) and the instrument response function (red) at 298 K, including the residuals (λ_{exc} = 376 nm, λ_{em} = 460 nm). Right: Fitting parameters including pre-exponential factors and confidence limits.

Fluorescence Spectra

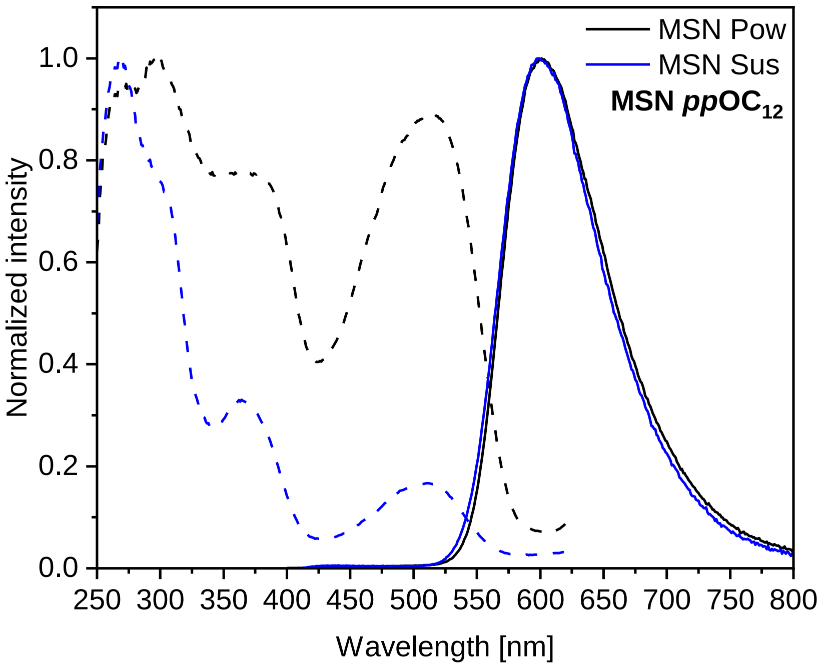

Figure S30: Excitation (dashed line, emission monitored at 640 nm) and emission (solid line, excitation at 300 nm) spectra of the MSNs with ***pp*OC$_{12}$** luminophore as powder (Pow, black) and in aqueous suspension (Sus, blue).

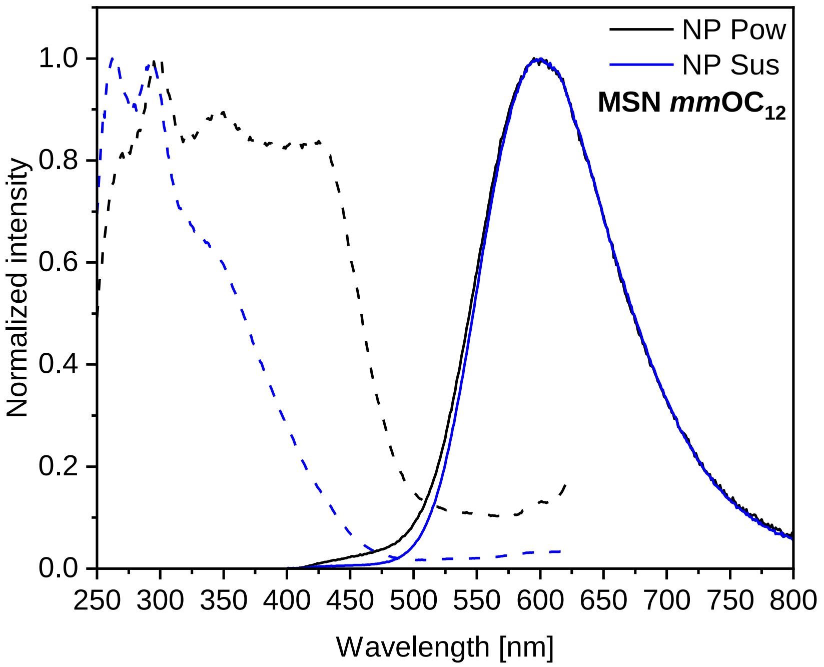

Figure S31: Excitation (dashed line, emission monitored at 640 nm) and emission (solid line, excitation at 300 nm) spectra of the MSNs with ***mm*OC$_{12}$** luminophore as powder (Pow, black) and in aqueous suspension (Sus, blue) before irradiation with UV light.

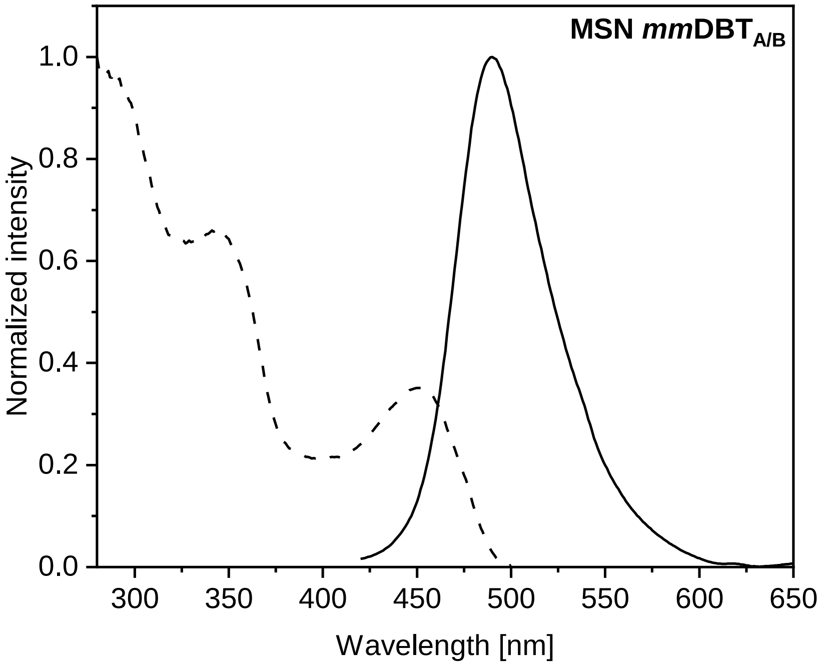

Figure S32: Excitation (dashed line, emission monitored at 520 nm) and emission (solid line, excitation at 345 nm) spectra of irradiated (365 nm, >30 min) MSNs with ***mm*OC$_{12}$** luminophore.

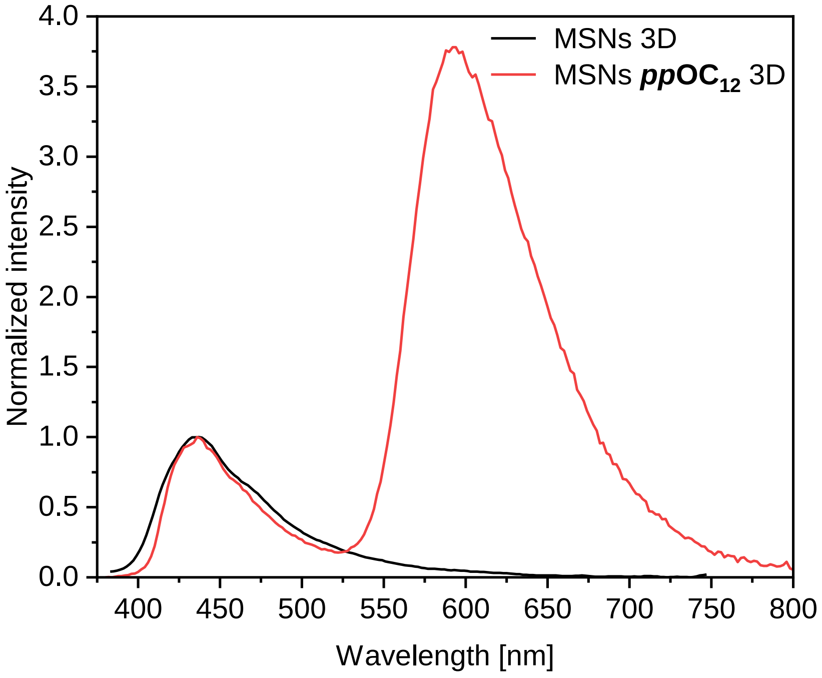

Figure S33: Emission spectra of 3D objects with MSNs with ***pp*OC$_{12}$** (red) and with MSNs without luminophore (black) measured with 375 nm excitation.

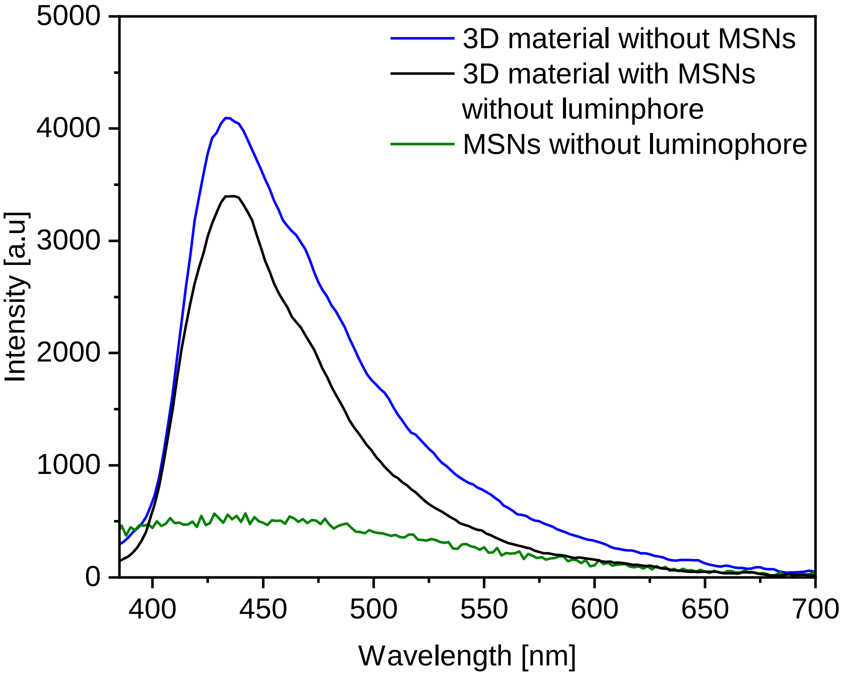

Figure S34: Emission spectra of MSNs without luminophore (red), 3D material without MSNs (black) and 3D material with MSNs without luminophore (blue) measured with 375 nm excitation.

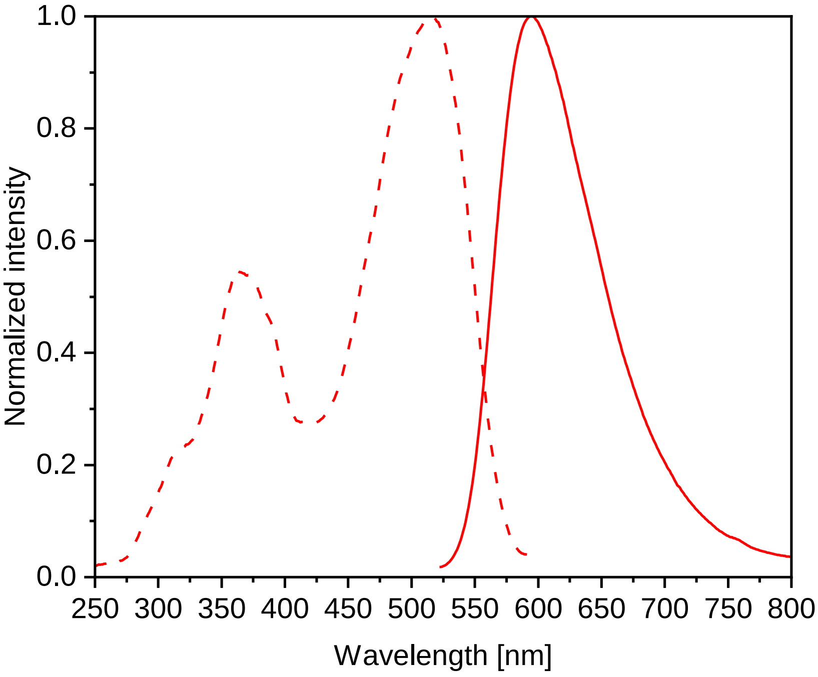

Figure S35: Emission (solid line, 510 nm excitation) and excitation spectrum (dashed line, emission monitored at 620 nm) of ***pp*OC$_{12}$** MSNs in 3D objects.

Irradiation Experiments

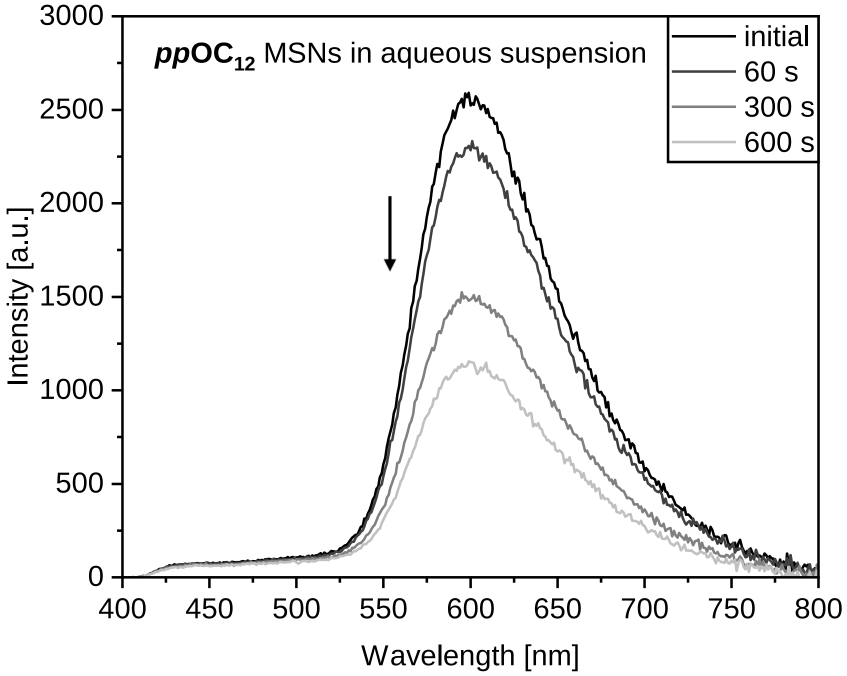

Figure S36: Emission spectra of MSNs with ***pp*OC$_{12}$** in aqueous suspension measured with 298 nm excitation. The particle suspension was irradiated outside the spectrometer with a 1.36 W 365 nm LED lamp for the stated amount of time.

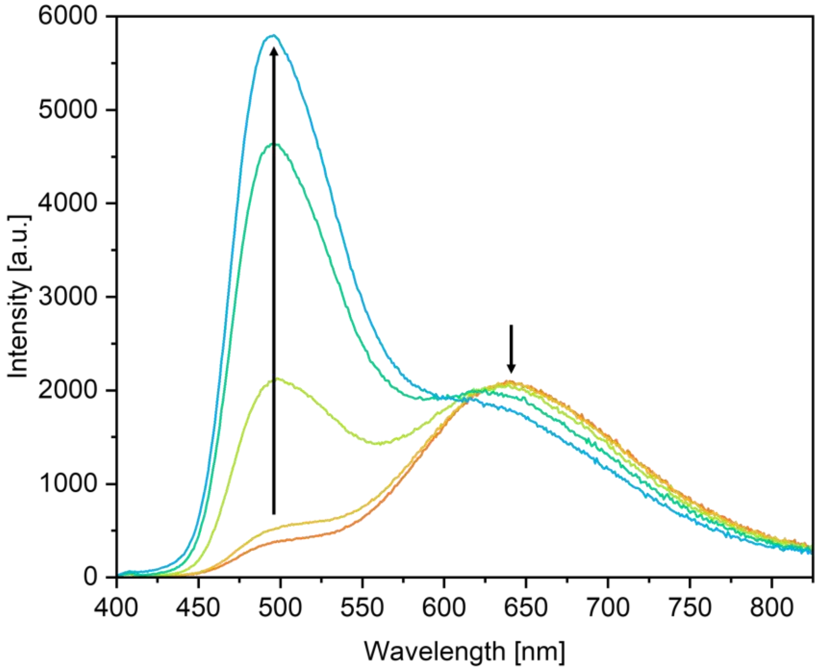

Figure S37: Consecutively measured emission spectra of ***mm*OC$_{12}$** in dichloromethane with 365 nm excitation.

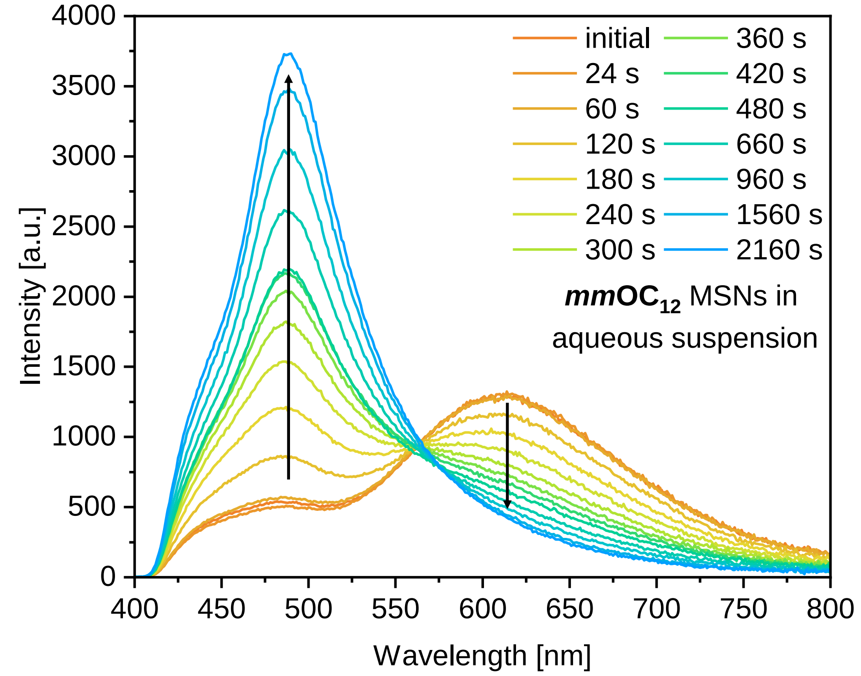

Figure S38: Consecutively measured emission spectra of the ***mm*OC$_{12}$** MSNs in aqueous suspension with 300 nm excitation.

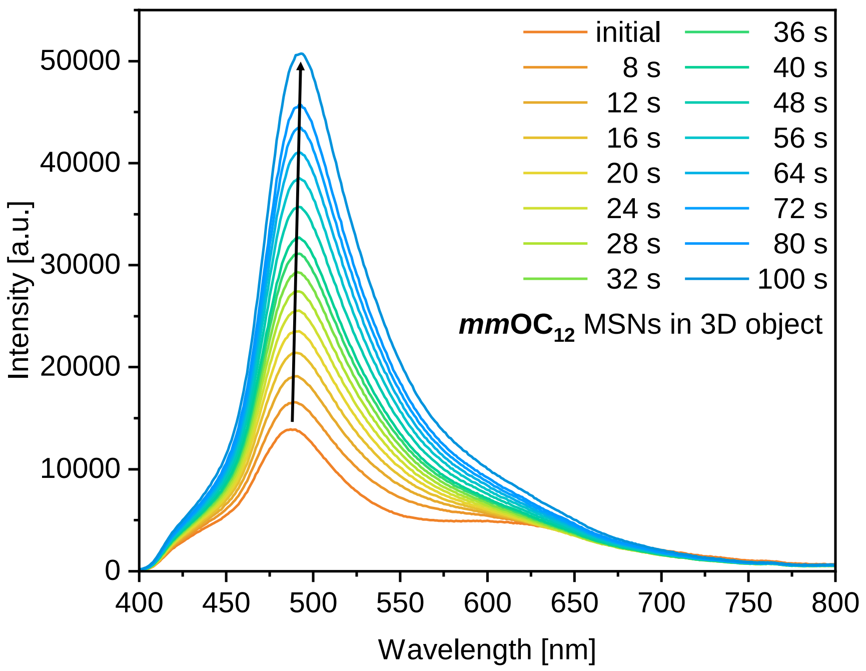

Figure S39: Consecutively measured emission spectra of the 3D material with ***mm*OC$_{12}$** MSNs with 365 nm excitation.

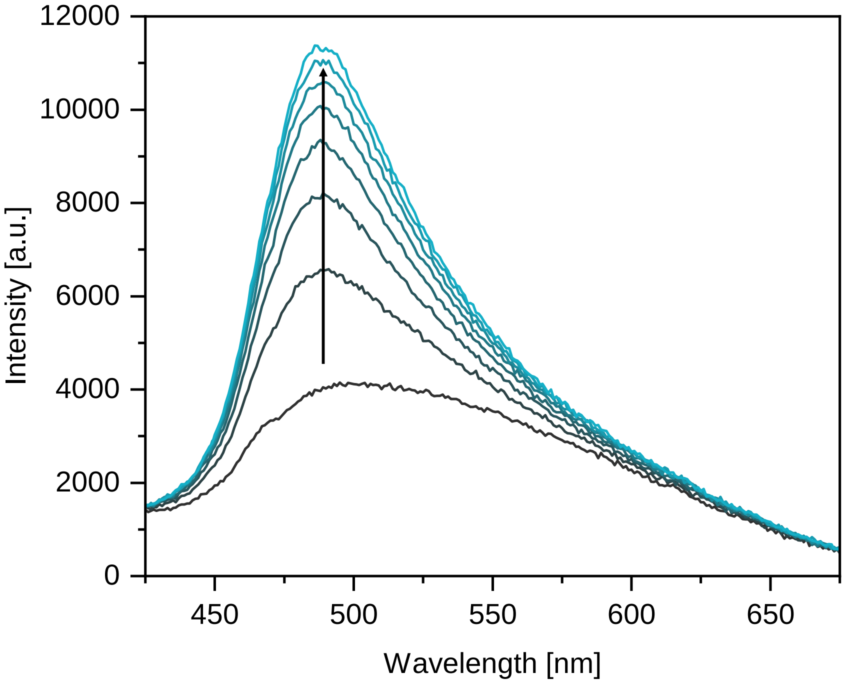

Figure S40: Consecutively measured emission spectra of the NFs with ***mmOC*$_{12}$** MSNs measured with 350 nm excitation.

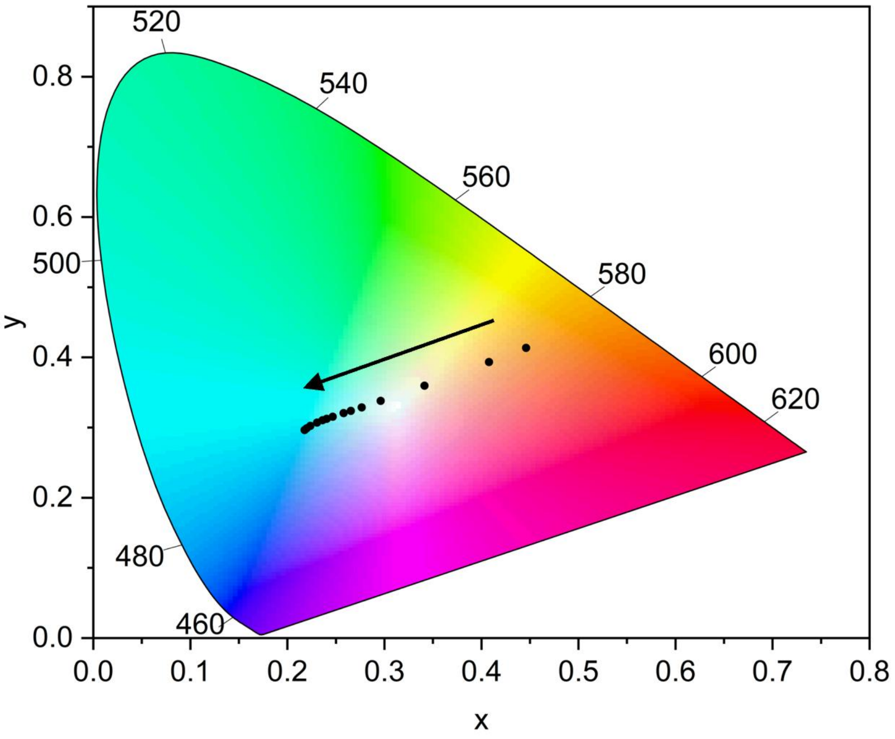

Figure S41: CIE plot of the MSNs with ***mmOC*$_{12}$** as powder. The points indicate the optical impression of the consecutive measured emission spectra (photocyclisation, Fig. 2D).

Mass Spectra

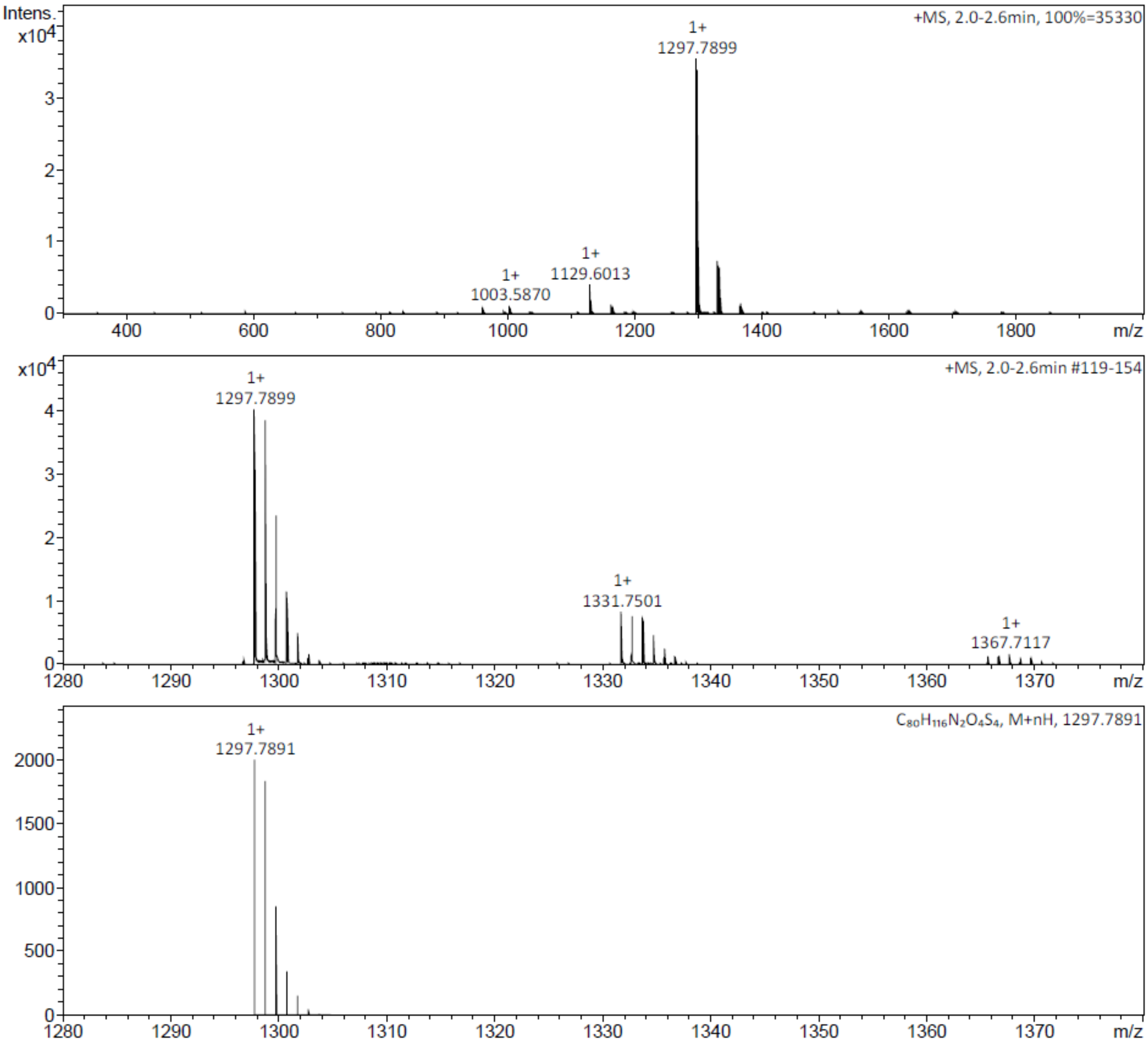

Figure S42: High-resolution mass spectrum of ***mm*OC$_{12}$** and simulated spectra (bottom) of [***mm*OC$_{12}$**+H]$^+$ before irradiation with UV light (cf. figure S43).

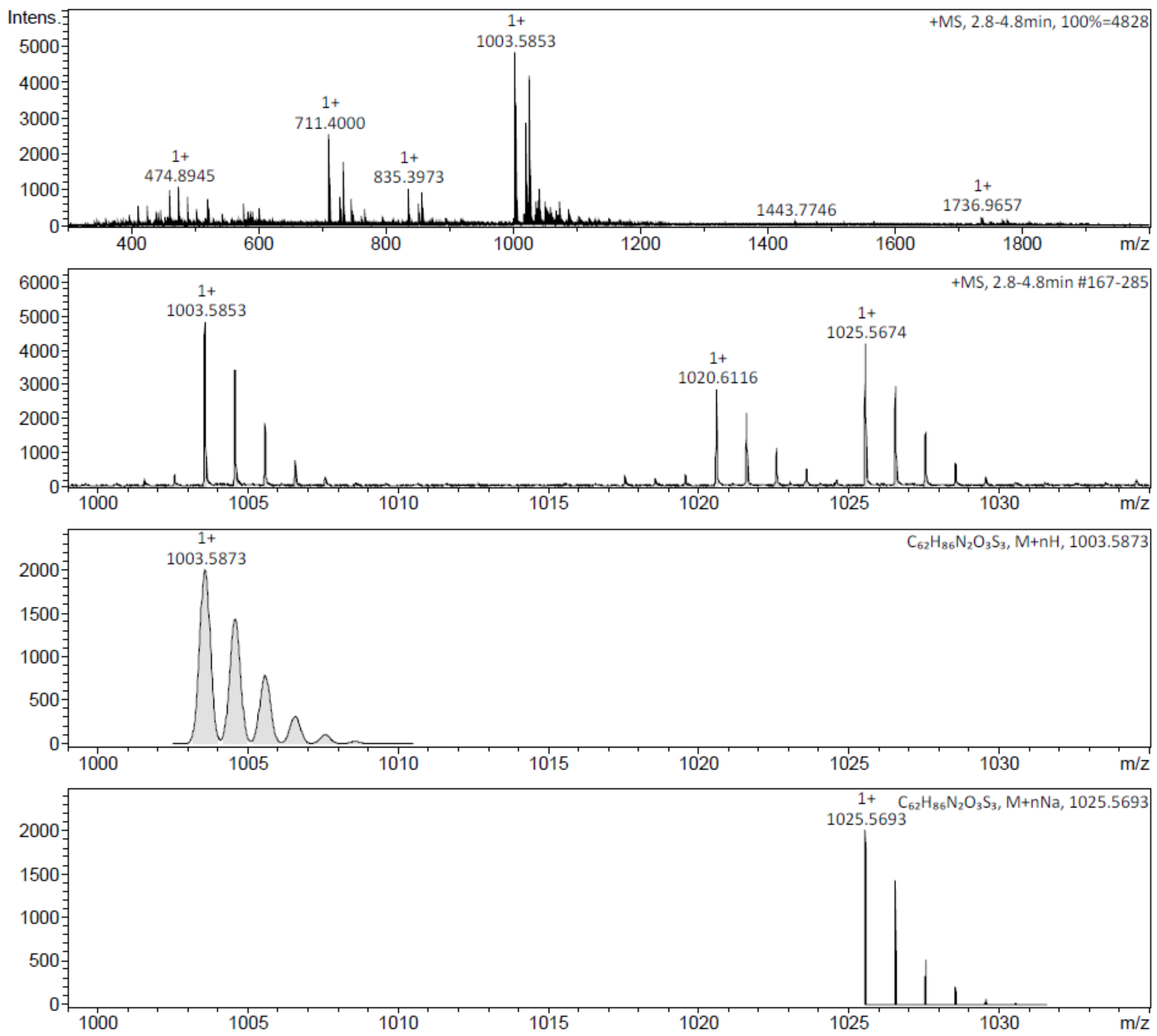

Figure S43: High-resolution mass spectrum of ***mm*****DBT**$_{\mathbf{A/B}}$ and simulated spectra (bottom) of [***mm*****DBT**$_{\mathbf{A/B}}$+H]$^+$ and [***mm*****DBT**$_{\mathbf{A/B}}$+Na]$^+$. The mass spectrum was obtained by irradiating a sample of ***mm*****OC**$_{\mathbf{12}}$ in dichloromethane in a mass vial with a 365 nm hand lamp for two minutes.

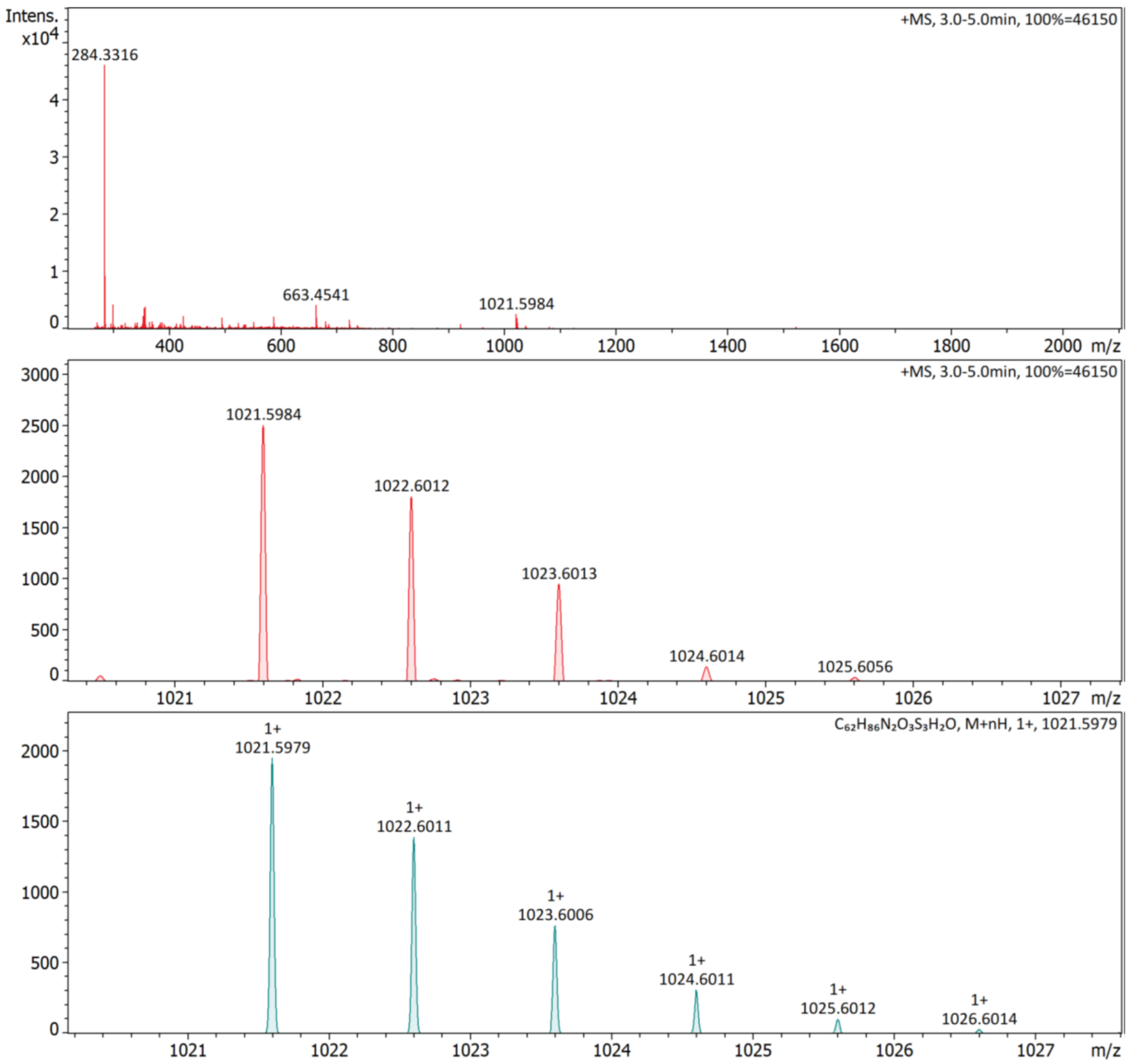

Figure S44: High-resolution mass spectrum of ***mm*DBT$_{A/B}$** extracted from the MSNs with DCM after irradiation with UV light (red) and simulated spectra (green) of [***mm*DBT$_{A/B}$**+H_2O+H]$^+$.

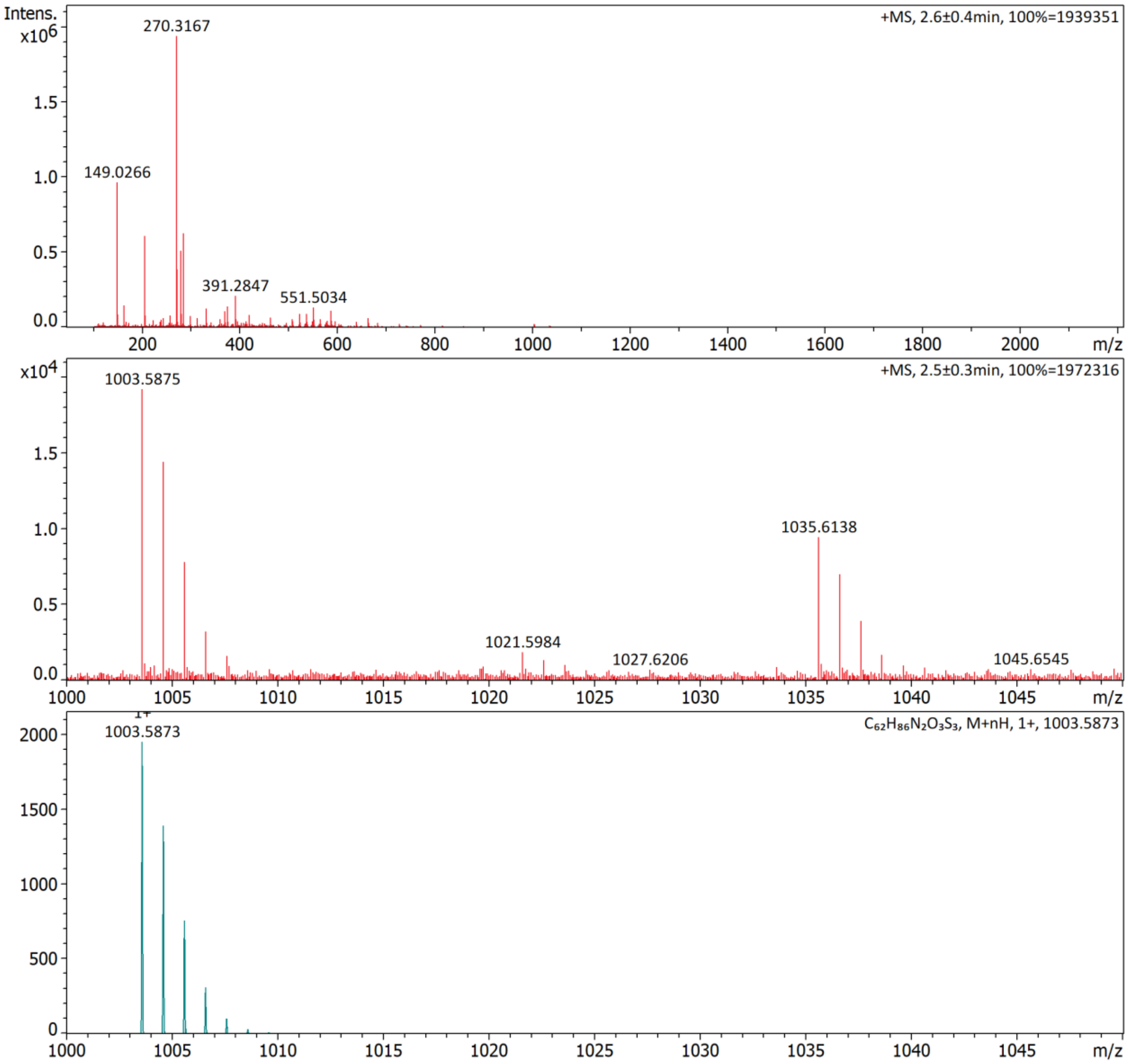

Figure S45: High-resolution mass spectrum of ***mm*DBT$_{A/B}$** extracted from the NFs with MSN with DCM after irradiation with UV light (red) and simulated spectra (green) of [***mm*DBT$_{A/B}$**+H]$^+$.

References

[1] S. Riebe, C. Vallet, F. van der Vight, D. Gonzalez-Abradelo, C. Wölper, C. A. Strassert, G. Jansen, S. Knauer, J. Voskuhl, *Chem. Eur. J.* **2017**, *23*, 13660.
[2] D. D. Lasic, *Angew. Chem.* **1994**, *106*, 1765.
[3] L. Donato, Y. Atoini, E. A. Prasetyanto, P. Chen, C. Rosticher, C. Bizzarri, K. Rissanen, L. De Cola, *Helv. Chim. Acta* **2018**, *101*, e1700273.
[4] S. Besson, T. Gacoin, C. Ricolleau, C. Jacquiod, J.-P. Boilot, *J. Mater. Chem.* **2003**, *13*, 404.
[5] W. Stöber, A. Fink, E. Bohn, *J. Colloid Interface Sci.* **1968**, *26*, 62.
[6] R. S. Fernandes, I. M. Raimundo, M. F. Pimentel, *Colloids Surf., A* **2019**, *577*, 1.
[7] A. Sillen, Y. Engelborghs, *Photochem. Photobiol.* **1998**, *67*, 475.
